# Experience sampling reveals habitual and transdiagnostic properties of compulsivity

**DOI:** 10.1038/s41398-026-04350-6

**Published:** 2026-07-31

**Authors:** Vanessa Teckentrup, Celine A. Fox, Kelly R. Donegan, Eoghan Gallagher, Anna K. Hanlon, Naomi A. Fineberg, Ilenia Pampaloni, Laura A. Berner, Claire M. Gillan

**Affiliations:** 1 School of Psychology and Trinity College Institute of Neuroscience, Trinity College Dublin, Dublin 2, Ireland; 2 FutureNeuro, the Research Ireland Centre for Translational Brain Science, Trinity College Dublin, Dublin 2, Ireland; 3 Department of Psychology, University of York, York, UK; 4 School of Life and Medical Sciences, University of Hertfordshire, Hatfield, UK; 5 South West London and St George’s Mental Health NHS Trust, London, UK; 6 Department of Psychiatry, Icahn School of Medicine at Mount Sinai, New York, USA; 7 Center for Computational Psychiatry, Icahn School of Medicine at Mount Sinai, New York, USA; 8 Global Brain Health Institute, Trinity College Dublin, Dublin 2, Ireland

**Keywords:** Human behaviour, Psychiatric disorders

## Abstract

Both obsessive thoughts and an urge to act have been linked to compulsions cross-sectionally. However, prominent theories of compulsivity disagree quite fundamentally about whether compulsions are goal-oriented attempts to reduce or eliminate obsessive thoughts or are urge-driven and habitual. To shed light on this debate, we used multilevel vector autoregression modelling to analyse contemporaneous and temporal associations based on self-report symptoms and cognition from *N* = 179 individuals with and without diagnoses who performed compulsions. Six weeks of three-times daily timeseries data with a mean lag of five hours between assessments were gathered from a transdiagnostic sample who checked their phone compulsively, binge ate, or engaged in compulsions commonly seen in OCD (e.g. checking, cleaning). Across compulsions, the strongest within-person correlate of compulsions were urges (β = 0.61, p_FDR_ < 0.001) and this was 3 times larger than obsessive thoughts and 4 times larger than anxiety. We found that urges predicted compulsions at the next assessment time point (β = 0.09, p_FDR_ < 0.001), while compulsions themselves had a markedly low tendency to predict themselves (β = 0.04) compared to the other mental health symptoms sampled (0.12 < β < 0.23). Finally, like habits, compulsions occurred more often when executive functioning was low (β = 0.09, p_FDR_ < 0.001). Based on real-time dynamics of compulsions, we find that urges, but not obsessive thoughts, predict compulsivity five hours later. Intriguingly, the associations were remarkably similar across compulsions, supporting the notion that transdiagnostically, a habit-like mechanism may underlie compulsive actions.

## Introduction

Compulsions are repetitive behaviours or mental acts that an individual feels driven to perform, even though those behaviours may conflict with high-level goals or serve no adaptive function [1, 2]. Although a transdiagnostic construct [3, 4], compulsivity is perhaps best exemplified by obsessive-compulsive disorder (OCD). The Diagnostic and Statistical Manual (DSM) defines compulsions in OCD as behaviours that arise either in response to an obsession, or according to rules that must be applied rigidly [2]. This presents a causal view of the genesis of compulsions that is in line with cognitive behavioural accounts of OCD, which suggest that obsessions, distressing and repetitive thoughts, and emotion states like anxiety give rise to compulsions [5]. Compulsive behaviours are thus framed as maladaptive, but nonetheless goal-oriented attempts to reduce or eliminate distressing thoughts and emotions. A similar framework is used to explain drug addiction, where it has been suggested that excessive valuation of drugs drives compulsive craving [6]. The habit hypothesis offers an alternative view, which suggests compulsions are better understood as automatic, stimulus-response behaviours [7], that are driven by external cues triggering urges, and can be carried out independent of their outcomes [8]. This perspective goes further to suggest that, in OCD, compulsions might even cause obsessions to develop as a *post hoc* explanation for behaviour or to reduce the cognitive dissonance that compulsive urges might generate [9, 10].

Considerable evidence for the habit hypothesis comes from cross-sectional studies using simple learning tasks where new stimulus-response behaviours are trained in the lab and then later tested for sensitivity to outcome value [11, 12]. In such studies, compulsive individuals often show the hallmarks of habit [13–15]: stimulus-driven behaviours that persist when they no longer serve a purpose [16]. In addition to this, studies have shown that a critical cognitive capacity that is important for curtailing habits [17, 18], goal-directed (or ‘model-based’) decision-making, is compromised across the compulsivity spectrum [19–24]. In lab settings, healthy individuals who repeatedly perform previously meaningless behaviours often begin to experience subjective urges to perform them [25, 26]. These premonitory urges or uncomfortable sensations, that even arise in the absence of any specific distressing worries or thoughts [27–30], mirror urges qualitatively described by OCD patients in their everyday lives [31]. This phenomenon appears across clinical and non-clinical populations—for example, patients with binge-eating disorder describe strong urges that precede binge episodes [32], as do normative samples who engage in compulsive phone-checking [33]. Although suggestive, empirical research, conducted in the real world, is needed to develop our understanding of the putatively habit-like properties of compulsions and understand how they relate to other disabling symptoms like anxiety and obsessions.

Ecological momentary assessment (EMA), which involves dense and repeated sampling of symptoms in real life, has become an increasingly popular approach in psychological sciences. The timeseries data generated through this method can allow us to examine how compulsions relate to emotions, thoughts, and cognitive functions within-person. It can also point to mechanisms potentially driving compulsivity by assessing temporal precedence using the Granger causality framework [34]. Taking this approach, the present study gathered six weeks of three-times daily EMA data from a transdiagnostic sample who checked their phone compulsively, binge ate, or engaged in one of a selection of compulsive behaviours commonly seen in OCD (e.g. checking, cleaning). By recruiting three groups with distinct manifestations of compulsivity, we aimed to test the extent to which the phenomenology generalises beyond diagnostic boundaries. Specifically, we hypothesised that compulsions in real life would be more strongly linked to urges than other candidate precursors like anxiety or obsessions. Consistent with this, the strongest within-person correlate of compulsivity was the experience of urges and this was three times larger than that of obsessive thoughts and five times larger than anxiety. Moreover, only urges and attempts to resist performing an action temporally preceded compulsive action. In an exploratory analysis, we found that compulsions had remarkably low autocorrelation compared to all other EMA items, which may be consistent with the idea that compulsions are phasic, reactive, and primarily driven by cues. Using a passively generated measure of cognitive processing speed [35], we tested if compulsivity increases when cognitive functioning is reduced. Similar to what has been shown for habits [36], we found that compulsions are performed more when cognition is compromised. For all these analyses, we found that although some small differences emerged, the findings were overall strikingly consistent across the different forms of compulsivity studied.

## Materials and methods

### Participants

We recruited individuals with OCD compulsions in collaboration with Hertfordshire Partnership NHS Foundation Trust (Hertfordshire, UK; *N* = 16) and South West London and St George’s Mental Health NHS Trust (London, UK; *N* = 4), and recruited individuals with eating compulsions in collaboration with the Icahn School of Medicine at Mount Sinai (New York, USA; *N* = 37). Additionally, *N* = 54 individuals were recruited from an existing database as they previously participated in research on OCD compulsions and had consented to being contacted for future studies [23]. Individuals interested in taking part in the study were screened using the Mini-International Neuropsychiatric Interview [37] and could proceed to the data collection phase if they fulfilled the criteria for obsessive-compulsive disorder, binge-eating disorder or bulimia nervosa. Furthermore, individuals with OCD compulsions completed the Yale-Brown Obsessive Compulsive Scale (YBOCS) [38], while individuals with eating compulsions completed a YBOCS version that was modified for binge eating (YBOCS-BE) [39]. Both versions of the YBOCS were conducted as interviews by trained members of the research team. Participants were instructed to download the smartphone research app ‘Neureka’ and to complete the ‘Pattern Wise’ module, a digital tracker assessing moment-to-moment experiences of an individually chosen compulsive behaviour three times a day over 6 weeks. Information on the compulsive behaviour patients chose to track and other compulsions patients reported to experience (but did not necessarily track) are shown in supplementary Table S13. Patients in the UK were paid £40 and patients in the US were paid $50 for participating in the study. Additionally, we included citizen scientists (*N* = 2105) who voluntarily subscribed to ‘Pattern Wise’ without remuneration and chose to track one of either traditional OCD behaviours (this included checking locks, cleaning, checking appliances, and counting), eating, or phone-checking. For this group of participants, we did not conduct clinical interviews. However, *N* = 3 citizen scientists who chose to track their eating behaviour, reported that they had received clinical treatment for their eating behaviour in the past but not at the time of the study. None of the citizen scientists who tracked OCD behaviours had ever received clinical treatment for their selected compulsion.

To be included for analyses, participants had to be at least 18 years of age and had to have a minimum of 50% of the ‘Pattern Wise’ assessments (N_assessments_ = 63) completed. Within the sample of individuals with a diagnosis, 56 individuals (OCD behaviours: *N* = 39, mean YBOCS = 20.7 ± 4.9; binge eating: *N* = 17, mean YBOCS-BE = 16.6 ± 5.6) fulfilled the inclusion criteria and a total of 123 participants (OCD behaviours: *N* = 24, binge eating: *N* = 35, phone checking: *N* = 64) without a diagnosis (citizen scientist sample) were included after the inclusion check. We provide detailed information on participant demographics, diagnoses, and treatments in Table 1. Please refer to Fig. S5 for a flowchart of the recruitment process. Among the included participants, individuals with a diagnosis who were paid for participation in the study completed 86.2 ± 11.6% of the assessments (OCD behaviours: 86%, binge eating: 86.5%) and individuals without a diagnosis who were unpaid completed 72.6 ± 14.8% of the assessments (OCD behaviours: 75.2%, binge eating: 74.3%, phone checking: 70.7%). For comparisons of the phenomenology of compulsions between participants with a diagnosis and those without, please see Fig. S8 in the online supplement. All participants gave written informed consent for their data to be included in analyses. The study was conducted in accordance with the relevant guidelines and regulations, including the Declaration of Helsinki, and approved by the research ethics committee of the School of Psychology, Trinity College Dublin (approval number: SPREC012021-12). Data collected through the Neureka app is stored and processed in line with the EU General Data Protection Regulations.

**Table 1:** Demographics for all compulsive behavior types and information on diagnoses, medication and psychotherapy for participants with a diagnosis.

		OCD behaviours		Binge eating		Phone checking
		Diagnosed *N* = 39	No diagnosis *N* = 24	Diagnosed *N* = 17	No diagnosis *N* = 35	*N* = 64
**Demographics**						
Age (*p* = 0.002**)		40.6 ± 12.7	61.5 ± 8.8	26.5 ± 4.4	49.8 ± 12.2	51.7 ± 13.8
Gender (*p* = 0.002**)		26 female 11 male 2 non-cisgender	17 female 7 male	14 female 2 male 1 non-cisgender	31 female 4 male	37 female 27 male
Education (*p* = 0.16)		2.9	2.8	3.1	3.0	2.7
**Diagnosis**						
Major Depressive Episode	Current	6		3		
	Past	31		15		
Major Depressive Disorder	Current	5		3		
	Past	27		10		
Manic Episode	Current	0		0		
	Past	2		1		
Hypomanic Episode	Current	0		0		
	Past	0		1		
Bipolar I Disorder	Current	0		0		
	Past	2		0		
Bipolar II Disorder	Current	0		1		
	Past	0		1		
Bipolar Disorder Unspecified	Current	0		0		
	Past	0		0		
Bipolar I With Psychotic Features	Current	0		0		
	Past	0		0		
Panic Disorder	Current	11		1		
	Past	14		4		
Agoraphobia	Current	9		2		
Social Anxiety Disorder	Current	2		5		
Obsessive-Compulsive Disorder	Current	39		2		
Posttraumatic Stress Disorder	Current	2		1		
Alcohol Use Disorder	Past 12 Months	2		1		
Substance Use Disorder	Past 12 Months	0		1		
Psychotic Disorder	Current	1		0		
	Lifetime	1		0		
Mood Disorder With Psychotic Features	Current	0		0		
	Lifetime	0		0		
Anorexia Nervosa	Current	0		0		
Bulimia Nervosa	Current	0		10		
Binge Eating Disorder	Current	2		7		
Generalised Anxiety Disorder	Current	15		8		
**Medication**						
SNRI	Current	0		2		
SSRI	Current	16		5		
Atypical antidepressant	Current	3		1		
Tricyclic antidepressant	Current	1		0		
Stimulant	Current	3		1		
Antipsychotic	Current	5		1		
Others	Current	14		4		
**Psychotherapy**						
CBT	Current	6		10		
DBT	Current	2		1		
Other	Current	2		1		

We compared mean age, gender distribution, and educational attainment between the three compulsive behavior types, averaging across individuals with and without a diagnosis within one behavior type. Diagnoses as displayed in the Table were assessed in a screening interview based on the Mini-International Neuropsychiatric Interview (MINI-7) [37] conducted by trained members of the research team. Information on medication and psychotherapy are based on participant self-report and were acquired during the same interview.

### Experimental procedure

**Fig. 1:**
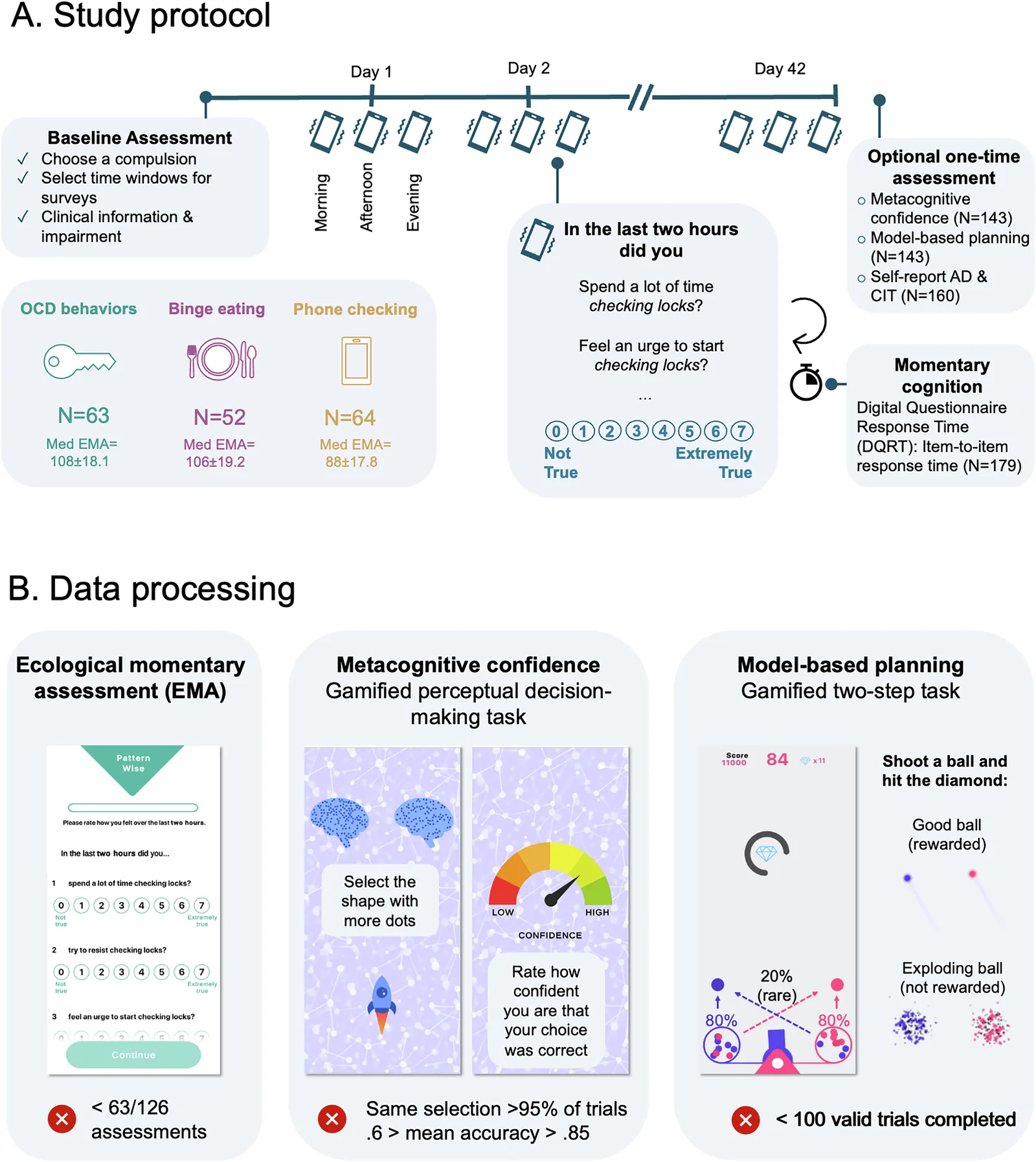
Study protocol and data processing. **A**. Participants experiencing one of three types of compulsive behaviour (OCD behaviours, binge eating, or phone checking) are recruited to complete six weeks of three-times-daily smartphone-based ecological momentary assessment (EMA). First, participants select which compulsion they want to track and the times for the three daily assessments that suit them best. After completing the EMA component, participants could choose to additionally complete a set of questionnaires used to calculate the transdiagnostic dimensions anxious-depression (AD) and compulsivity and intrusive thoughts (CIT), a perceptual decision-making task measuring metacognitive confidence, and a two-step task measuring model-based planning. Using EMA item-to-item response times, we further calculated a passive measure of cognitive processing speed (Digital Questionnaire Response Time, DQRT). **B**. To be included in the study, participants had to complete a minimum of 63 (50%) out of the total 126 EMA assessments. Task-based data went through a pre-processing step to ensure sufficient quality of the data. Participants were free to engage with the tasks for as many runs as they wanted. For analyses, we included the first valid and complete run per participant. For our gamified perceptual decision-making task, we excluded participants who had selected the left or right shape in more than 95% of the trials. Furthermore, we excluded participants with an accuracy outside of the pre-defined range between 0.6 and 0.85 as this was suggestive of a failure of the staircase procedure designed to ensure that confidence estimates are not confounded by interindividual differences in performance. For our gamified two-step task, we excluded participants who had not completed a full run of 100 trials.

Participants downloaded the smartphone research app ‘Neureka’ and completed the sign-up process which included questions pertaining to their demographics such as age, gender, and education level. After sign-up, individuals with a diagnosis were instructed to start the ‘Pattern Wise’ module and complete two short games (Cannon Blast and Meta Mind) testing facets of cognition which have previously been linked to compulsive behaviours (Fig. 1A). In contrast, citizen scientists were free to explore ‘Neureka’, and we only included individuals who chose to complete ‘Pattern Wise’. A subset of these individuals also chose to complete one or both cognitive games. However, this was not required to be included in the analyses.

### Modules

#### Pattern wise – momentary compulsive experiences

The Pattern Wise module is an ecological momentary assessment (EMA) tool that follows participants for 42 days and asks them to rate their momentary compulsive experiences three times a day (Fig. 1B). Upon starting the module, participants first choose the behaviour they want to track from a list of pre-specified options or selecting ‘other’ to track an individually defined behaviour. Participants then complete four items assessing the clinical relevance of their compulsions before entering the EMA phase of the module. Here, participants receive notifications three times per day to complete 11 items assessing their compulsive experiences over the last two hours. Items were presented in a fixed order and participants responded using an 8-point Likert response scale, which numerically ranged from zero to seven. The first 8 items are unipolar and assess the time spent doing the individually chosen compulsion (referred to as ‘compulsivity’ in the results section), if the participant tried to resist (try to resist), if they felt an urge to start the compulsive behaviour (urge), if they felt in control over their compulsions (feel in control), if they had repetitive distressing thoughts (obsessive thoughts), if they experienced stressful events (stressful events), and if they felt bored (bored) or guilty (guilty). The remaining 3 items are bipolar and ask for participants’ affect on a continuum from happy to sad (happy), energetic to tired (tired), and relaxed to anxious (anxious). The full wording of the EMA items and anchors displayed for the response scale can be found in Table 2. The three assessments per day were always spaced five hours apart but participants could customize the set of assessment times to best accommodate their daily-life habits. Once a week, participants further completed an adapted version of the Work and Social Adjustment Scale [40] that considered their chosen compulsion, which was not included in the analyses reported here.

**Table 2:** Ecological momentary assessment (EMA) items and anchors displayed for the response scale.

Experience	Question Text Prompt: In the last two hours…	Left Anchor	Right Anchor
Compulsivity	…did you spend a lot of time [chosen compulsion]?	Not true	Extremely true
Try to resist	…did you try to resist [chosen compulsion]?	Not true	Extremely true
Urge	… did you feel an urge to start [chosen compulsion]?	Not true	Extremely true
Feel in control	… did you feel in control over [chosen compulsion]?	Not true	Extremely true
Obsessive thoughts	… did you have repetitive, distressing thoughts?	Not true	Extremely true
Stressful events	… did you experience stressful events?	Not true	Extremely true
Bored	…did you feel bored?	Not true	Extremely true
Guilty	…did you feel guilty?	Not true	Extremely true
Happy	… did you feel sad or happy?	Sad	Happy
Tired	…did you feel energetic or tired?	Energetic	Tired
Anxious	…did you feel relaxed or anxious?	Relaxed	Anxious

At the top of the screen the prompt “In the last two hours…” was displayed which was followed by the EMA items. All items were displayed on the same page as a vertical list where the list would auto-scroll once the participant had responded to an item. Participants could scroll through the list to return to previously answered items. Participants responded using an 8-point Likert response scale, which numerically ranged from zero to seven. The left anchor was displayed at the left end of the scale (zero), and the right anchor was displayed at the right end of the scale (seven). Once all items were answered, participants had to confirm their responses by pressing a button at the bottom of the screen.

#### Transdiagnostic dimensions – self-report questionnaires

A subset of participants (*N* = 160; N_OCD_ = 61, N_Binge eating_ = 47, N_Phone checking_ = 52; N_with diagnosis_ = 54; M_age_ = 48.8 ± 15.0; M_YBOCS_ = 20.6 ± 5.0; M_YBOCS-BE_ = 16.6 ± 5.6) completed 49 items selected from six self-report questionnaires assessing depression (SDS; Zung Self-Rating Depression Scale) [41], trait anxiety (STAI; State Trait Anxiety Inventory) [42], impulsivity (BIS-11; Barratt Impulsiveness Scale) [43], obsessive–compulsive disorder (OCI-R; Obsessive–Compulsive Inventory–Revised) [44], eating disorders (EAT; Eating Attitudes Test) [45], apathy (AES; Apathy Evaluation Scale) [46]. This set of items has been validated [47] against the original set of 209 items [48] used to derive transdiagnostic factors of psychopathology that summarize the item-level responses. Applying the factor weights from Wise and Dolan [47] to the item-level responses yields two transdiagnostic factors reflecting anxious-depression and compulsivity and intrusive thought.

#### Gamified cognitive tasks

##### Meta mind (Perceptual decision-making task) – metacognitive confidence bias

Meta Mind is a gamified version of a perceptual decision-making task [49] assessing an individuals’ ability to reflect upon their own cognitive ability (Fig. 1B). Among other measures, the task allows to derive an individuals’ metacognitive bias, a measure of their average level of confidence in their own choices in a difficult perceptual decision-making problem. Previous work has repeatedly shown that individuals scoring higher in compulsive traits tend to report higher confidence in their own choices whereas individuals high in anxiety and depression are underconfident [50–52]. A detailed description and validation of Meta Mind has previously been published [53] and further information is available in the online supplement. In their screening sessions, participants with a diagnosis were instructed to complete Meta Mind as a one-time assessment in the Neureka app while citizen scientists freely explored the app. Still, completing Meta Mind was not required for inclusion in the analysis and a subset of *N* = 143 (N_OCD_ = 58, N_Binge eating_ = 41, N_Phone checking_ = 44; N_with diagnosis_ = 54; M_age_ = 49.0 ± 15.4; M_YBOCS_ = 20.6 ± 5.0; M_YBOCS-BE_ = 16.6 ± 5.6) out of the full 179 participants had valid data on their metacognitive bias.

##### Cannon blast (Two-Step Task) – model-based planning

Cannon Blast is a gamified version of the two-step reinforcement learning task [17] which estimates a participant’s ‘model-based’ tendency, representing the extent to which they make decisions using a mental map of action-outcome associations (Fig. 1B). Previous work has shown that compulsivity is linked to a reduction in model-based decision-making [24, 48], hence, boosting the tendency to follow habits in individuals high in compulsivity [7]. A detailed description and validation of Cannon Blast has previously been published [21] and further information is available in the online supplement. In their screening sessions, participants with a diagnosis were instructed to complete Cannon Blast as a one-time assessment in the Neureka app while citizen scientists freely played this game. Again, completing Cannon Blast was not required for inclusion in the analysis and a subset of *N* = 143 (N_OCD_ = 49, N_Binge eating_ = 41, N_Phone checking_ = 53; N_with diagnosis_ = 41; M_age_ = 48.5 ± 14.8; M_YBOCS_ = 20.7 ± 5.4; M_YBOCS-BE_ = 16.4 ± 6.0) out of the full 179 participants had valid data on their tendency to make model-based choices.

#### Digital questionnaire response time – cognitive processing speed

We used a recently validated passive measure to assess a third facet of cognitive ability, cognitive processing speed. Digital Questionnaire Response Time (DQRT, 35) uses item-level timestamps to derive item-to-item response times which have been shown to be linked to traditional task-based measures of processing speed. Here, we averaged across the 11 EMA items presented at each assessment timepoint in the ‘Pattern Wise’ module to derive momentary measures of cognitive processing speed that we used to quantify moment-to-moment change in cognition. By taking the mean DQRT across all assessments within-person, we further looked into group-level differences in cognitive processing speed which have been shown to be linked to differences in compulsivity in previous work [54, 55]. A detailed description of DQRT is available in the online supplement. As DQRT is based on the EMA items, all *N* = 179 participants also had valid data for analyses using DQRT.

### Data analysis

All data were pre-processed and analysed using R Statistical Software (v4.4.0 [55]). Data, code and materials for this research are available at https://osf.io/s2z7n/. To get access to the data, researchers are required to submit a Data Request Form which is linked in the OSF repository.

#### EMA data preparation

To facilitate model estimation, we added a small amount of random noise drawn from a uniform distribution between −0.1 and +0.1 to each EMA variable. This ensured that models could be estimated even if participants repeatedly selected the same value for an EMA variable, thus, reducing the variability. EMA data can contain trends that violate the assumption of stationarity. We tested each time series across all 11 EMA items for stationarity using the Kwiatkowski–Phillips–Schmidt–Shin (KPSS) test. A majority of the time series (65%) was stationary [56], so we did not additionally detrend the EMA time series. Longitudinal DQRT is known to be subject to a training effect, meaning that DQRT reduces over time following a negative exponential curve [35]. In line with this, only a small fraction of the DQRT time series (19%) were stationary. To remove these and lower order trends from DQRT, we ran a regression model predicting longitudinal DQRT by assessment day (the number of days elapsed from the start of Pattern Wise) to model the linear trend and assessment day raised to the power of −0.2 to model the negative exponential trend. We then added the residuals from this model to the intercept to obtain detrended DQRT values.

For all models, continuous variables used as predictors were scaled and mean-centred across the sample except for models based on longitudinal data where predictors were centred within-participant to improve model convergence. The group variable reflecting the three behaviour types was implemented as a factor variable with contrast coding with the phone-checking group defined as the base level. The factor variable was set up to have a mean value of zero and a standard deviation of one, thus, bringing it in line with the scaled continuous predictor variables.

#### Between-person analyses of group differences

To assess differences between the three compulsive behaviour types on the between-person level, we ran ANOVAs that included group (OCD behaviours, binge eating, phone checking) as a predictor and age, gender, and education as covariates. Post-hoc tests were run using the multcomp [57] package in R. Resulting p-values were corrected for multiple comparisons across EMA variables and pairwise comparisons between the groups by controlling the false discovery rate (FDR) using the Benjamini-Hochberg method [58]. This type of model was used to assess group differences in transdiagnostic dimensions, metacognitive bias, model-based planning, mean cognitive processing speed and variance in EMA items over time.

#### Univariate analyses on data acquired through repeated assessments

Our main goal was to identify mechanisms driving compulsive action. Hence, the EMA item assessing time spent on performing the individually chosen compulsion was our main variable of interest which we termed ‘compulsivity’ and used as the dependent variable in most models using EMA data. All models were based on the implementation of multilevel vector autoregression models as outlined in the R package ‘mlVAR‘ [59] but modified to run univariate analyses using lme4 [60] that allowed to include covariates. A sensitivity analysis using a hurdle model to address zero-inflation in our main outcome variable, compulsivity, is described in the supplement.

To test for potential directed effects of each of the 10 EMA variables on compulsivity, we ran time-lagged models which are a method of establishing temporal precedence, often called ‘Granger causality’. A variable ‘Granger-causes’ a target variable if its past values contain information to predict the target variable above and beyond the information contained in the target variable alone [34]. Importantly, temporal precedence is a necessary but not sufficient condition for establishing causality as unmeasured variables could be driving changes in both observed variables [61]. As such, Granger causality only points to *potential* causes of an observed effect as it likely that potentially relevant latent variables (such as differences in the temporal profiles of different psychological experiences or environmental triggers or cues) were not captured. As fixed effects, each model included the time-lagged time series of the respective EMA variable and the time-lagged time series of compulsivity as a predictor. The latter was added to isolate the unique contribution of each EMA variable’s time course above and beyond the autocorrelation of compulsivity. The mean across time within each participant (excluding the mean of the dependent variable) was included as a predictor to estimate between-person effects. We added interaction terms with group (OCD behaviours, binge eating, phone checking), age, gender, and education for both predictor time series. The interaction term with group was submitted to further analysis using the marginaleffects [62] R package to extract group-specific estimates and all pairwise comparisons between the three behaviour types. Pairwise comparisons were corrected for multiple comparisons within the marginaleffects function by controlling the FDR. The remaining interaction terms were added to account for covariates of no interest. On the second (participant) level, we modelled both random intercepts and random slopes for the time-lagged EMA and compulsivity predictors. Hence, the full equation for the last assessment time point (t-1) of one of the EMA variables (in this example ‘urge’) predicting the current assessment time point (t) of compulsivity was: Compulsivity_t_ ~ (Urge_t-1_ + Compulsivity_t-1_) * (Group + Age + Gender + Education) + Mean(Urge) + (1|ID) + (0 + Urge_t-1_|ID) + (0 + Compulsivity_t-1_|ID). We ran the same model with compulsivity and each of the 10 remaining EMA variables as dependent variables to test for temporal precedence in both directions (EMA predicting compulsivity and compulsivity predicting EMA). Following the established two-step method of mlVAR, we then estimated within-person contemporaneous associations between compulsivity and each of the 10 remaining EMA variables using the residuals extracted from the directional models estimated in step one. Then, we averaged the estimates for how well each of the EMA-related residuals predicted the compulsivity residuals (Compulsivity_residuals_ ~ 0 + (Urge_residuals_ * Group) + (0 + Urge_residuals_|ID)) and how well the compulsivity residuals predicted each of the EMA-related residuals (Urge_residuals_ ~ 0 + (Compulsivity_residuals_ * Group) + (0 + Compulsivity_residuals_|ID)). Again, we added an interaction term for group to the residual models to extract group-specific estimates and all pairwise comparisons between the three behaviour types for the contemporaneous associations. As we had longitudinal assessments of DQRT (cognitive processing speed), we used the same type of directional models to assess time-lagged effects of DQRT on all EMA variables (including compulsivity) and vice versa, before estimating the contemporaneous associations using the residuals extracted from the directional models.

To quantify autocorrelation which reflects the tendency of each EMA variable to predict its own future values, we ran separate models which predicted each of the EMA time courses by the respective time-lagged EMA variable. We added the same interaction terms and again modelled a random intercept and random slope for the time-lagged EMA variable.

Finally, we tested if individual difference measures such as symptom severity as measured by the transdiagnostic dimensions (anxious-depression (AD), compulsivity and intrusive thought (CIT)), or task-based cognition (metacognitive confidence and model-based planning) modulated any of the contemporaneous, time-lagged or autocorrelative effects. For these models, we added an interaction term to the fixed effects that reflected the interaction between the predictor time-series and the respective individual difference measure. As the transdiagnostic dimensions are known to share variance, we ran AD and CIT in the same model while we set up separate models for each cognitive task measure.

All results reported are corrected for multiple comparisons across EMA variables by controlling the FDR.

#### Multivariate network models

One limitation of traditional vector autoregressive models used for assessing temporal precedence is their reliance on bivariate associations that ignore interactions with other measured time series which can lead to confounded inferences [63]. Hence, we ran multivariate vector autoregressive model, also known as network models, which take all associations between EMA variables into account at once and return partial correlations between variables, corrected for all other associations [64]. Network models can suffer from model instability if the proportion of data points missing within the time series is too substantial. To test if our dataset contained enough information for each participant to obtain stable network outcomes, we ran a network stability test. For this, we first calculated temporal network estimates (i.e. the partial correlations describing each bivariate time-lagged association while accounting for all other associations) for all 11 EMA variables (yielding 121 estimates per participant) based on the full available data. Next, we iteratively dropped 5% of the datapoints from each individual participant’s time series until reaching 85% of the data dropped. Across each of the nine steps we calculated the temporal network estimates again. Then we calculated the within-person correlation between network estimates derived from the full time series and the network estimates derived from the down-sampled data across each step. Finally, we identified the maximum proportion of the data that can be dropped to still retain a correlation of r = 0.7 between the network estimates derived from the full time series and those derived from the down-sampled data. This correlation stability coefficient should assume values above 0.5 and should not fall below 0.25 [65].

We used mlVAR [59] implemented in R to run the multivariate vector autoregression model with all 11 EMA variables. We specified a lag of 1, defined an ID variable to identify repeated assessments belonging to one individual and assigned a beep variable that identified the assessment time point each data point belonged to, thus, identifying missing assessments. All other settings were kept on the mlVAR defaults. We then extracted the temporal, or directed, network estimates to assess potential drivers of compulsive actions as identified through multivariate modelling.

## Results

### The trait and state phenomenology of OCD compulsions is near indistinguishable from binge-eating and phone-checking compulsions

**Fig. 2:**
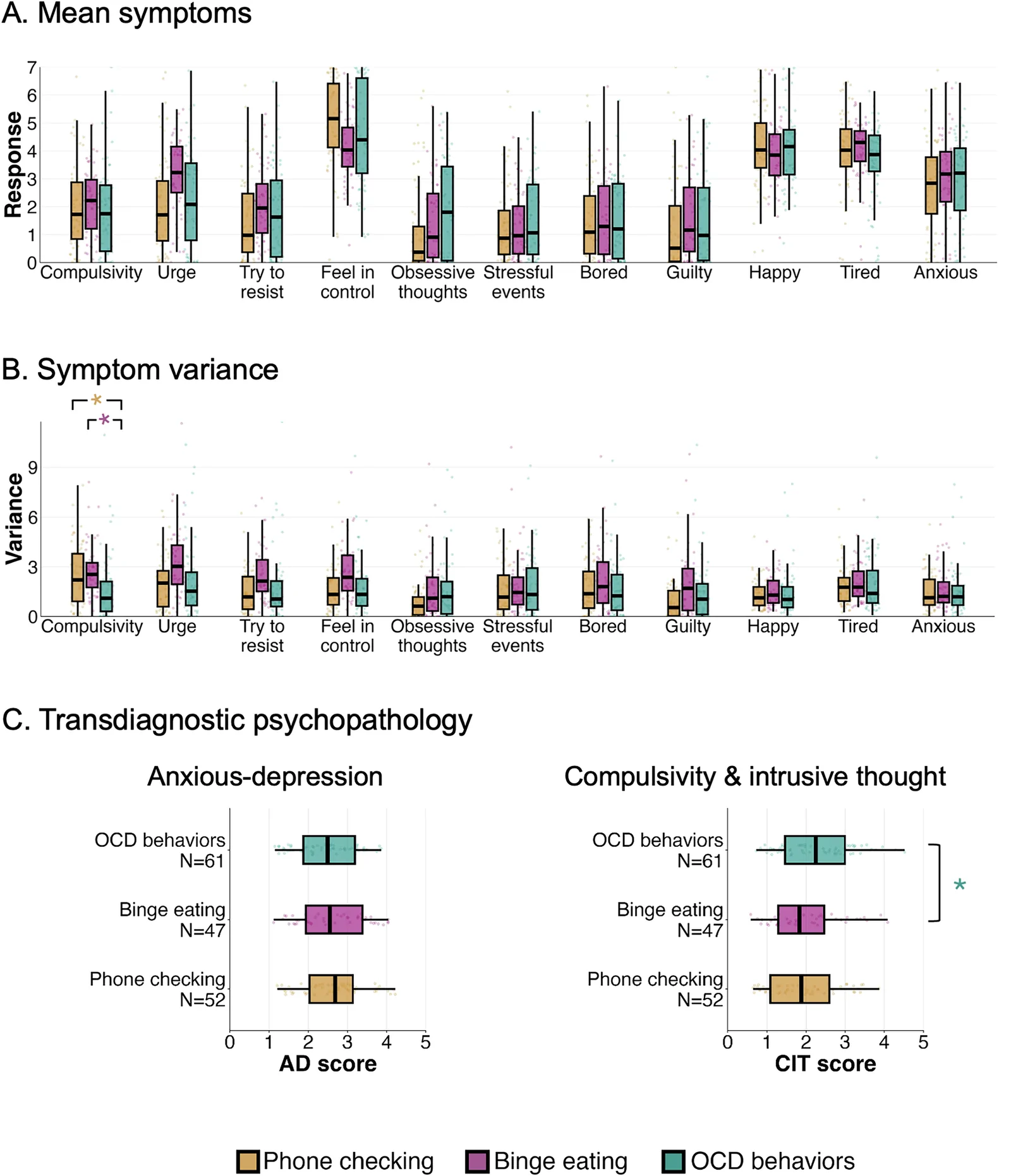
At both state and trait levels, the phenomenology of compulsions is preserved across compulsive behaviour types. **A**. Mean symptom levels measured across 11 ecological momentary assessment items show no statistical difference between any of the three compulsive behaviour types. **B**. In line with this, only variance in compulsivity was higher for the binge-eating and the phone-checking group compared to the OCD-behaviour group. We found no differences for variance in symptom levels between the compulsive behaviour types across the remaining 10 ecological momentary assessment items. **C**. Transdiagnostic dimensions of symptom severity only differed for compulsivity & intrusive thought (right panel) where the OCD-behaviour group reported higher severity levels compared to the binge-eating group. There were no group differences for anxious-depression. In all panels, coloured dots show individual-level data averaged across all available assessments within person while boxplots summarize the data across participants with the box outlines depicting the interquartile range and the line in the middle depicting the median. Annotation: * = *p* < 0.05; ** = *p* < 0.01; *** = *p* < 0.001.

First, we tested if our three behaviour types—OCD compulsions, eating compulsions and phone-checking compulsions—were comparable in the amount of time spent performing them (i.e., their compulsivity). We found no difference between groups in the mean of self-reported compulsivity over the 6 weeks of the study (p_FDR_>0.05). Next, we tested for differences in the 10 other psychological states assessed via EMA: urges to perform the compulsion, efforts to resist, degree of control over the compulsion, having obsessive thoughts, experiencing stressful events, boredom, guilt, happiness, tiredness and anxiety. There were no group-differences in mean symptom levels in any of the EMA items (all p_FDR_ > 0.05; Fig. 2A). Only for compulsivity, variance across assessments was lower for the OCD-behaviours group compared to binge-eating (Δβ = −0.98, SE = 0.32, p_FDR_ = 0.042) and phone-checking (Δβ = −0.93, SE = 0.30, p_FDR_ = 0.042). We found no differences in variance between the groups for the remaining 10 EMA items (all p_FDR_ > 0.05; Fig. 2B). Turning to self-reported trait levels of transdiagnostic psychopathology, the groups did not differ in their ‘anxious depression’ (AD) levels (minimum p_FDR_ = 0.39), but the OCD-behaviour group reported higher ‘compulsivity and intrusive thought’ (CIT) compared to the binge-eating group (difference=0.44, SE = 0.16, p_FDR_ = 0.02; Fig. 2C).

### Urge is the strongest within-person correlate of time spent performing compulsions in all three groups

**Fig. 3:**
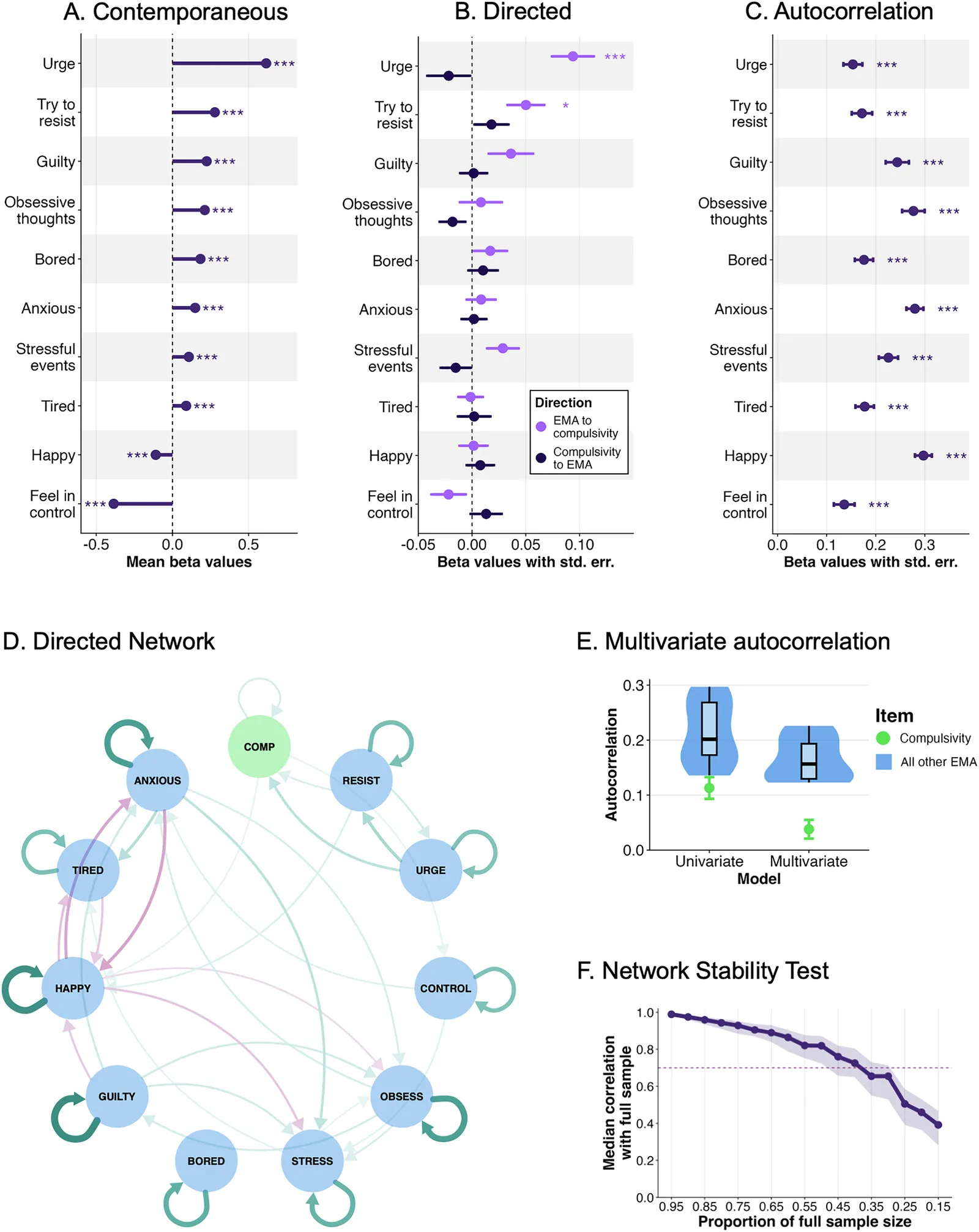
Urges and attempts to resist the compulsion show the largest association with compulsivity. Univariate models assessing the **A**. contemporaneous association between compulsivity (time spent on the compulsion) and the remaining ten psychological experiences indicate urges as the biggest contributing factor, with its effect size exceeding that of obsessive thoughts three-fold and anxiety four-fold. **B**. Running directed (time-lagged) associations with either psychological experiences predicting compulsivity at the next time point or vice versa, reveal that only urges and attempts to resist predict future compulsivity with no evidence for reverse Granger causation. **C**. All psychological experiences showed significant autocorrelation. However, compulsivity had the lowest autocorrelation followed by feelings of being in control, urges, and attempts to resist compulsions. **D**. A multivariate model, that takes associations between all ecological momentary assessment (EMA) variables into account at once, confirms urges and attempts to resist compulsions as the main predictors of compulsivity. Again, obsessive thoughts and feelings of anxiety did not show a significant directed relationship with compulsivity. Interestingly, the autocorrelation of compulsivity is strikingly low, even though compulsive action appears to be driven by behavioural states with comparingly higher tendencies to predict themselves (urges and attempts to resist compulsions). **E**. Indeed, compulsivity showed the lowest autocorrelation out of all EMA variables in both the multivariate and, to a lesser degree, in the univariate model. **F**. As directed multivariate network models require a high number of datapoints within-person to achieve stable outcomes, we ran a network stability test wherein we iteratively drop data points within-person in increments of 5% up to 85% of the data dropped and compare the network structure derived from the unaltered time series to the network structure derived from the down-sampled data. The stability coefficient for networks derived from the current data was 0.6, meaning that 60% of the data can be dropped (or only 40% of the data points need to be preserved) to still retain a correlation of r = 0.7 (red dashed line) between the networks derived from the full time series and the down-sampled time series. Annotation: * = *p* < 0.05; ** = *p* < 0.01; *** = *p* < 0.001 Network model nodes: COMP: compulsivity (time spent performing compulsions); RESIST: try to resist; URGE: urge, CONTROL: feel in control; OBSESS: obsessive thoughts; STRESS: stressful events; BORED: bored; GUILTY: guilty; HAPPY: happy; TIRED: tired; ANXIOUS: anxious.

We ran linear mixed-effects models to test how within-person fluctuations in the 10 psychological states were linked to compulsivity in the three types of compulsions. Compulsivity was contemporaneously associated with all ten states (positively-coded states: −0.11<mean β < −0.39, negatively-coded states: 0.09<mean β < 0.61; all p_FDR_ < 0.001, Fig. 3A). The strongest link was observed for urges (β = 0.61), and this was true for all three groups (OCD behaviours: β = 0.70; binge eating: β = 0.52; phone checking: β = 0.63). Repetitive obsessive thoughts (β = 0.21) and anxiety (β = 0.15) were considerably weaker correlates, with the effect size of urge exceeding obsessive thoughts three-fold and exceeding anxiety four-fold. The weakest within-person correlates of compulsivity were feeling tired (β = 0.09) and experiencing stressful events (β = 0.11).

Next, we tested for differences across the three behaviour types. Seven between-group differences in these contemporaneous associations survived FDR correction, out of the 30 tested (supplementary Fig. S1 and Table S1). The association between urge and compulsivity was stronger in the OCD-behaviour group compared to binge-eating (Δβ = 0.18, p_FDR_ = 0.048). The OCD-behaviour group had a stronger link between compulsivity and obsessive thoughts than the binge-eating group (Δβ = 0.25, p_FDR_ = 0.004), while stressful events were more strongly linked to compulsivity in the OCD-behaviour group compared to binge-eating (Δβ = 0.16, p_FDR_ = 0.03) and phone-checking (Δβ = 0.14, p_FDR_ = 0.049). The phone-checking group had a stronger link between boredom and compulsivity than the binge-eating (Δβ = 0.21, p_FDR_ = 0.001) and OCD-behaviour groups (Δβ = 0.27, p_FDR_ < 0.001). Finally, the OCD-behaviour group had a stronger link between compulsivity and anxiety compared to the binge-eating group (Δβ = 0.17, p_FDR_ = 0.03).

### Urges and attempts to resist are the only psychological states that significantly predicted future compulsivity

We tested if any of the ten EMA items predicted compulsivity at the next assessment point, over and above the information contained in the autocorrelation of compulsivity. This is a method for establishing temporal precedence in observational time series data. Strikingly, only urges (β = 0.09, SE = 0.02, p_FDR_ < 0.001) and attempts to resist (β = 0.05, SE = 0.02, p_FDR_ = 0.02) predicted the future performance of compulsions (Fig. 3B). A sensitivity analysis based on a hurdle model to address zero-inflation in the compulsivity time-series further substantiated these findings. Again, we found for the zero-inflation component, predicting the probability of observing a zero compared to any non-zero value, that only urges and attempts to resist predicted a higher probability to observe a non-zero value for compulsivity at the next time point (supplementary Table S12). There were no differences between the groups in the strengths of these predictive relationships (supplementary Fig. S2 and Table S2), and we did not find evidence for reverse temporal precedence for any EMA item (Fig. 3B), as there were no significant effects for the opposite direction across or between the groups (all p_FDR_ > 0.05).

### Compulsivity is characterised by low autocorrelation

While all EMA variables showed significant autocorrelation (all p_FDR_ < 0.001, Fig. 3C), we observed the lowest autocorrelation for compulsivity (β = 0.11, SE = 0.02) followed by feeling in control of compulsions (β = 0.14, SE = 0.02), urges to perform them (β = 0.15, SE = 0.02) and trying to resist them (β = 0.17, SE = 0.02). In comparison, the remaining states; that is, happiness, anxiety, obsessive thoughts, stress, guilt, tiredness and boredom showed higher estimates (0.18 < β < 0.30). There was only one difference between the three behaviour groups of 30 tested that survived FDR correction (supplementary Fig. S3 and Table S3); compulsivity had lower autocorrelation in the binge-eating group than the other two groups (phone checking: Δβ = 0.17, SE = 0.04, p_FDR_ = 0.001; OCD behaviours: Δβ = 0.13, SE = 0.04, p_FDR_ = 0.01). In fact, the autocorrelation was not statistically significant in the binge-eating group (β = 0.01, SE = 0.03, p_FDR_ = 0.71) while there was a positive autocorrelation for phone checking (β = 0.18, SE = 0.03, p_FDR_ < 0.001) and OCD behaviours (β = 0.14, SE = 0.03, p_FDR_ < 0.001). We ran a permutation test to confirm that all eleven EMA time series contained a meaningful temporal order to calculate autocorrelation from (supplementary Fig. S9).

### Multivariate (network) models show comparable findings

Next, to add context to our findings from the univariate approach, we carried out a multivariate analysis that takes contemporaneous and directed associations between all variables into account by calculating partial correlations (Fig. 3D). In this network analysis including all eleven EMA variables, compulsivity showed an even lower temporal autocorrelation (β = 0.04, SE = 0.02) compared to all other states (0.12 < β < 0.23; Fig. 3E).

### Within-person, slower cognitive processing speed is related to compulsive behaviour dynamics

**Fig. 4:**
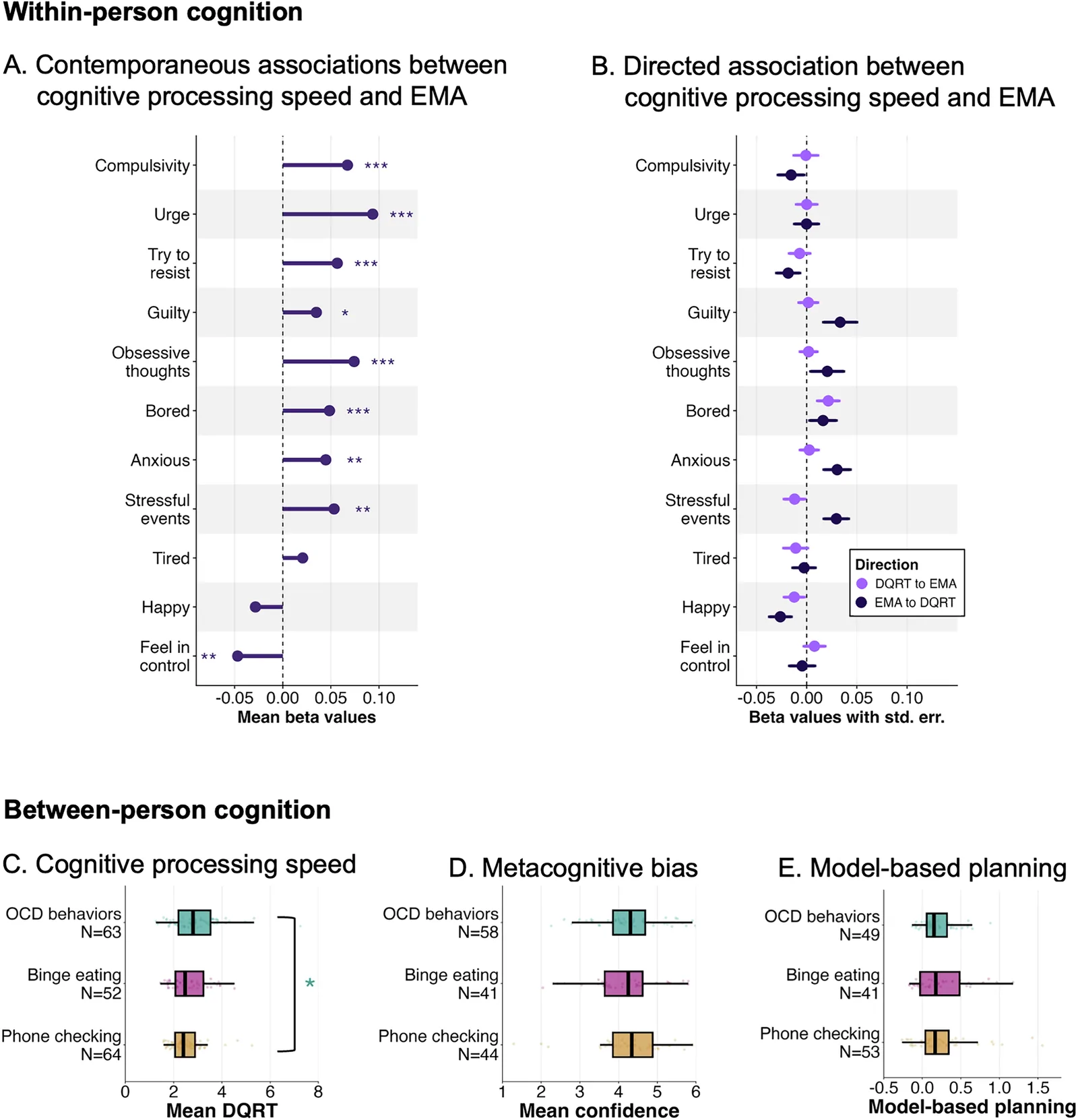
Within-person, slower cognitive processing speed is linked to higher compulsivity. **A** Contemporaneously, cognitive processing speed as measured by Digital Questionnaire Response Time (DQRT), a passive measure of cognitive processing speed based on survey item response times, is slower for worse scores in all ecological momentary assessment (EMA) variables except for feeling tired. **B** We did not find any significant associations for directed associations with either EMA variables predicting future DQRT or vice versa. **C** One-time assessment of mean cognitive processing speed was slower in the OCD-behaviour group compared to the phone-checking group. **D** In contrast, metacognitive bias shows no group-level differences. **E** Similarly, model-based planning does not differ between the three groups. Annotation: * = *p* < 0.05; ** = *p* < 0.01; *** = *p* < 0.001.

Next, we assessed how compulsivity related to within-person changes in cognition measured using Digital Questionnaire Response Time (DQRT), a passive measure of cognitive processing speed based on survey item response times [35]. Contemporaneously, all affective states and compulsive tendencies, except for tiredness and happiness, were linked to slower cognitive processing speed (positively-coded states: β = −0.05, negatively-coded states: 0.03<β < 0.09; all p_FDR_ < 0.05; Fig. 4A). The strongest effect was for cognitive processing speed to be slower at the same time as individuals experienced greater urges (β = 0.09, p_FDR_ < 0.001). This effect was twice the size of that for anxiety (β = 0.04, p_FDR_ = 0.01) but of a similar size as that for obsessive thoughts (β = 0.07, p_FDR_ < 0.001). There were no significant directed effects for DQRT and the EMA items in either direction (all p_FDR_ > 0.05; Fig. 4B). All relationships were consistent across the three behaviour types, with no significant differences observed (supplementary Fig. S4 and Table S4). In terms of individual differences, mean DQRT was slower in the OCD-behaviour group compared to the phone-checking group (Δβ = 0.43, SE = 0.15, p_FDR_ = 0.01; Fig. 4C), but there were no differences between the OCD-behaviour and binge-eating groups (Δβ = 0.20, SE = 0.16, p_FDR_ = 0.21) or between the binge-eating group and phone-checking group (Δβ = 0.22, SE = 0.17, p_FDR_ = 0.20). Clinical severity in terms of AD and CIT was not linked to mean DQRT (both *p* > 0.05).

### Between-person assessments of symptom severity and cognition do not show a significant association with real-world compulsive dynamics

In a final set of analyses, we were interested in whether there were individual differences in how compulsivity related to other symptoms. For example, if more anxious patients might have stronger links between anxiety and compulsivity. This could contribute to explaining the heterogeneity of clinical presentation within disorders of compulsion. To do this, we examined how transdiagnostic clinical scores of AD and CIT related to the EMA data. Higher compulsivity and intrusive thought was linked to increases in compulsivity (β = 0.21, SE = 0.07, p_FDR_ = 0.04), more urges (β = 0.19, SE = 0.07, p_FDR_ = 0.04) and higher stress (β = 0.18, SE = 0.07, p_FDR_ = 0.04). Higher AD, in contrast, was linked to increases in all EMA scores (0.24 < β < 0.41, all p_FDR_ < 0.01), except for trying to resist the compulsion and feeling in control. Against our prediction, neither CIT nor AD modulated the contemporaneous association between EMA scores and compulsivity (all p_FDR_ > 0.05). Neither dimension modulated the directed associations between EMA scores and compulsivity (all p_FDR_ > 0.05). In contrast, an increased autocorrelation for stressful events was associated with higher CIT (β = 0.07, SE = 0.02, p_FDR_ = 0.009) whereas higher AD was linked to higher autocorrelation for feeling bored (β = 0.07, SE = 0.02, p_FDR_ = 0.02).

Although the sample size was small, we also explored if task-based assessments of metacognitive confidence and model-based planning (gathered once at baseline) were related to differences in how compulsivity related to other EMA items. Neither metacognitive confidence (Fig. 4D) nor model-based planning (Fig. 4E) differed between groups (all p_FDR_ > 0.05). Moreover, there was no relationship between scores on either task and compulsivity (model-based planning: β = −0.06, SE = 0.08, p_FDR_ = 0.93; metacognitive confidence: β = 0.1, SE = 0.07, p_FDR_ = 0.62), or any other EMA item (all p_FDR_ > 0.05). Similarly, neither cognitive measure was associated with individual differences in the autocorrelation of compulsivity, or its contemporaneous or directed associations with EMA scores (all p_FDR_ > 0.05) with one exception—higher metacognitive confidence was linked to a stronger association between feeling happy and compulsivity at the next assessment (β = 0.03, SE = 0.01, p_FDR_ = 0.02).

## Discussion

There is considerable debate around whether compulsive actions are goal-oriented or habitual [66]. Cognitive theories suggest in addiction, compulsive behaviours are attempts to obtain a highly valued reward [6] and in OCD, compulsions are attempts to reduce distress caused by maladaptive cognitions and anxiety [5, 67]. The habit hypothesis proposes that compulsions arise from an over-activation of the brain’s habit system whereby external stimuli or contexts can trigger an urge to complete a response that is both irresistible and independent of current motivation [11, 12]. So far, evidence for the role of habit has predominantly come from lab-based studies of newly learnt behaviours. This controlled approach isolates general mechanisms that confer vulnerability to compulsivity, avoiding the confounds of well-established compulsive routines and associated cognitions. However, this gain in experimental control comes at the cost of ecological validity. Here, we took a different approach and used ecological momentary assessments to examine if real-life compulsions have habitual properties, and if the compulsivity phenomenon truly generalizes beyond diagnostic boundaries.

We found that compulsivity was strongly related to urges, far more than other experiences sampled, including obsessions or anxiety. This was true overall, and for each compulsion type individually. Furthermore, albeit with small effect sizes overall, only urges and attempts to resist the compulsion predicted compulsions at the next assessment, even while controlling for autocorrelation in the compulsivity time series. These findings add to a growing literature highlighting the role of urges as a separable, mechanistic factor in compulsive phenomenology [28]. In the present study, there was no evidence for reverse temporal precedence—that is, we did not find any evidence that performing compulsions predicted increases or decreases in future EMA items. However, it is important to note that our multi-hour timescale of investigation (three times a day with five hours between assessments) does not capture any dynamics of compulsivity that fluctuate on faster timescales. For example, one study measured urges in OCD patients at the level of seconds and found that when not suppressed, urges increased before performing mental compulsions and decreased following execution in around 60 s [27]. We also found that compulsions had remarkably low autocorrelation, particularly when we considered all other EMA items in our multivariate models. Again, the five-hour lag between assessments may have contributed to this low autocorrelation. Although we cannot fully address this without a more frequent sampling regime, in a supplementary analysis we showed that all autocorrelation coefficients were significantly larger than those calculated from a permuted time series. This indicates that even considering the five-hour lag, the EMA time series contained a meaningful temporal order to calculate autocorrelation from. This is further corroborated by autocorrelation estimates obtained from affective variables which despite the five-hour lag showed significant autocorrelation. To put this in context, the autocorrelation estimate for obsessive thoughts was twice and five times the size of that for compulsivity for the univariate and multivariate models, respectively. This observation is interesting but may not be particularly discerning of either framework – as goal-directed behaviour and habits alike might be expected to have low autocorrelation. What it does underscore, however, is that compulsions, unlike emotional states, do not appear to feed-forward onto themselves over time, or might be fluctuating on a timescale that differs from that of emotional states.

Experimental studies have shown that habits are more likely to be expressed under conditions where executive control is compromised, for example through cognitive load [36], stress [68] or in rodents, following targeted deactivations of goal-directed brain systems [69]. Here, we found naturalistic within-person data in line with this account. Participants spent more time performing compulsions and had stronger urges when their executive functioning was lower than usual, though overall the effects were non-specific as cognitive slowing was linked to increases in all negative states (except tiredness). Unlike a recent study (with a larger sample) that found that depression symptoms temporally predicted cognitive slowing using DQRT [70], here we did not find evidence for temporal precedence in either direction. In terms of cross-sectional cognition, studies have identified cognitive differences in individuals high in compulsivity, such as model-based planning [21] and metacognitive confidence [53]. One possibility is that these individual differences might map onto different manifestations of compulsivity. However, we did not find evidence that model-based planning or metacognitive confidence was linked to the severity of real-world compulsivity or any dynamic property of compulsivity studied using EMA. This could be due to low power. Previous work has shown reliable but small effect sizes for both associations [21, 53], requiring hundreds of participants.

Our findings were remarkably similar across the different compulsion types we studied. The DSM has begun to recognise that compulsivity is a transdiagnostic construct, acknowledging shared compulsive features in eating disorders or addiction disorders [71]. Compulsive phone-checking is not recognized by the DSM, but studies increasingly show it has substantial overlap with OCD symptomatology [3, 4]. It is important to note that citizen scientists represented the majority of our sample (123 out of 179 participants in total) which could suggest that the dynamics we report are more characteristic of subthreshold compulsive symptomatology. However, we did not find substantial differences in compulsivity-related dynamics between phone-checking, a group which fully consisted of citizen scientists (*N* = 64), and OCD behaviours, a group that included more individuals with a diagnosis (*N* = 39) than without (*N* = 24). The most substantial group-difference we found was a significantly lower autocorrelation for compulsivity in the binge-eating group. This might be due to differences in the phenomenology of binges which according to the diagnostic criteria are expected to occur less frequently (a minimum of 1 day per week) than OCD compulsions (taking up to an hour per day) [2]. Notwithstanding, it is striking that urges were the strongest correlate of compulsive behaviour in all three compulsive behaviour types.

This study had a number of limitations. One important point to consider is the influence that a sampling rate of three times per day with a lag of 5 h between assessments has on the present findings. According to the Nyquist theorem in signal processing [72], to correctly characterize the frequency of a fluctuating time series, the sampling rate needs to be twice the size of the highest frequency component in the signal. For depression, it has been suggested that one assessment per week could suffice [73]. In individuals without a diagnosis, one occurrence of intrusive thoughts was observed per two weeks [74]. Things get more complex, however, when one aims to map temporal relationships between variables. For instance, in an observational study of hand-washing behaviour between 7 and 38 hand-washing episodes occurred per day and urges were commonly reported only once, even if they were followed by multiple hand-washing attempts [75]. In addition, long-term prospective studies showed that OCD symptoms most often develop during adolescence [76], follow an intermittent course wherein obsessions and compulsions can change in both quantity and quality while clinically relevant symptoms are observable for decades [77]. Similarly, the causal structure underlying compulsive acts might change over time with stimulus-response associations linked to habits gaining relatively more weight compared to obsessions as drivers of compulsivity. For this reason, we see the present study as a starting point for future efforts to map out these relations for varying symptom durations and at both faster (minutes) and slower frequencies (months) to build robust and falsifiable models of these dynamics. More specifically, future studies could consider experimental designs that increase the probability of capturing clinically relevant events. This could be assessments that are presented at random times during the day or event-contingent sampling wherein participants actively report the occurrence of a compulsive behaviour instead of responding to a predefined assessment schedule. To reduce participant burden, for some compulsions it could be a promising approach to use passive measures for tracking compulsivity. For example, screen-on time can be tracked to objectively measure phone-use [78] while preliminary findings point to a potential use of continuous glucose monitors for tracking episodes of disordered eating [79]. Next, it should be noted that observational time-series data cannot establish causal relationships. The psychological processes assessed could still collectively be affected by external, unmeasured factors, making variables appear causally related. Similarly, the causal structure is likely to differ between individuals. Hence, for clinical translation, person-specific models—which for example can be implemented using temporal networks as well—are more appropriate. Still, approaches that rely on temporal precedence such as the one employed in this EMA study provide vital information on which mechanisms to target for future intervention-based research [80]. Finally, the citizen scientists we included in this study were older than the group of individuals with a diagnosis, because the neureka app user base tends to skew older. It is not possible to fully disentangle the potential confound between age and diagnosis, but sensitivity analyses support the idea that these groups were highly similar (supplementary Tables S14–S16). Interestingly, diagnosed OCD had stronger contemporaneous relationships between compulsivity and anxiety and between compulsivity and obsessions. This may match the clinical picture where diagnosis is met once the symptoms of compulsivity become distressing [2]. However, future work would need to interrogate this more directly in age-matched samples.

To conclude, we used EMA to robustly characterise the real-time dynamics of OCD, binge-eating, and phone-checking compulsions in daily life. Several observations were consistent with a role for habitual processes in compulsivity, most notably the importance of premonitory urges. While these data do not dispute the importance of maladaptive beliefs and anxiety in OCD and other compulsive disorders, they prompt a reconsideration of their moment-to-moment relevance for compulsive behaviour.

## Supplementary information

Supplementary Information

## Data Availability

Data, code and materials for this research are available at https://osf.io/s2z7n/. To get access to the data, researchers are required to submit a Data Request Form which is linked in the OSF repository.
