# Supplementary material for: Experience sampling reveals habitual and transdiagnostic properties of compulsivity
Source: Transl Psychiatry. 2026 Jul 31;16:591. doi: 10.1038/s41398-026-04350-6 (PMC13714317; doi:10.1038/s41398-026-04350-6)
Supplement: Supplementary file 1 [file 41398_2026_4350_MOESM1_ESM.docx]

**Experience sampling reveals habitual and transdiagnostic properties of compulsivity**

**Supplementary Information**

Vanessa Teckentrup^1,2*^, Celine A Fox^1^, Kelly R Donegan^1,3^, Eoghan Gallagher^1^, Anna K Hanlon^1^, Naomi A Fineberg^4^, Ilenia Pampaloni^5^, Laura A Berner^6,7^, & Claire M Gillan^1,2,8^

^1^	School of Psychology and Trinity College Institute of Neuroscience, Trinity College Dublin, Dublin 2, Ireland

^2^ FutureNeuro, the Research Ireland Centre for Translational Brain Science, Trinity College Dublin, Dublin 2, Ireland

^3^ Department of Psychology, University of York, York, United Kingdom

^4^ School of Life and Medical Sciences, University of Hertfordshire, Hatfield, UK

^5^ South West London and St George's Mental Health NHS Trust, London, UK

^6^ Department of Psychiatry, Icahn School of Medicine at Mount Sinai, New York, USA

^7^ Center for Computational Psychiatry, Icahn School of Medicine at Mount Sinai, New York, USA

^8^ Global Brain Health Institute, Trinity College Dublin, Dublin 2, Ireland

**Corresponding author***

Dr Vanessa Teckentrup, [vanessa.teckentrup@tcd.ie](mailto:vanessa.teckentrup@tcd.ie)

Trinity College Dublin, College Green, Dublin 2, Ireland

**TABLE OF CONTENTS**

[RESULTS	4](#_Toc230285081)

[Figure S1: Main effects per group and group comparisons for the contemporaneous associations between EMA and compulsivity.	4](#_Toc230285082)

[Table S1: Differences between the compulsive behavior types in contemporaneous associations between EMA and compulsivity (comp.).	5](#_Toc230285083)

[Figure S2: Main effects per group and group comparisons for time-lagged associations with EMA predicting compulsivity at the next time point.	7](#_Toc230285084)

[Table S2: Differences between the compulsive behavior types in time-lagged associations with EMA predicting compulsivity at the next time point.	8](#_Toc230285085)

[Figure S3: Main effects per group and group comparisons for the autocorrelation of EMA.	10](#_Toc230285086)

[Table S3: Differences between the compulsive behavior types in autocorrelation of EMA.	11](#_Toc230285087)

[Figure S4: Main effects per group and group comparisons for contemporaneous associations between EMA and cognitive processing speed as measured by DQRT.	13](#_Toc230285088)

[Table S4: Differences between the compulsive behavior types in contemporaneous associations between EMA and cognitive processing speed as measured by DQRT.	14](#_Toc230285089)

[Comparisons between individuals with a diagnosis and those without in the OCD behaviors and binge eating groups	16](#_Toc230285090)

[Figure S8: Across the OCD behaviors and binge eating groups, experiences associated with compulsions are more pronounced in individuals with a diagnosis.	17](#_Toc230285091)

[Assessing signal contained in the behavioral time-series data for autocorrelation analyses	19](#_Toc230285092)

[Figure S9: Ecological momentary assessment (EMA) time series with a five-hour lag between assessments contain sufficient information to calculate autocorrelation.	20](#_Toc230285093)

[Table S10: Demographics for non-completers of the Pattern Wise module who were not included in the main analyses, split by compulsive behavior type and diagnostic status.	21](#_Toc230285094)

[Figure S11: Response distributions for each of the 11 ecological momentary assessment variables by compulsive behavior types.	22](#_Toc230285095)

[Sensitivity analysis addressing zero-inflation in the compulsivity outcome through a hurdle model	23](#_Toc230285096)

[Table S12: Comparison between the outcomes of a multilevel vector autoregressive model (mlVAR) reported in the main text and a hurdle model which addresses zero-inflation in our main outcome variable ‘compulsivity’.	25](#_Toc230285097)

[Table S13: Compulsive behaviors participants selected to track in the neureka app and other compulsions patients reported to experience but did not track in the app.	26](#_Toc230285098)

[Table S14: Differences between participants in the OCD behaviors group with and without a diagnosis in contemporaneous associations between EMA and compulsivity (comp.).	28](#_Toc230285099)

[Table S15: Differences between participants in the OCD behaviors group with and without a diagnosis in time-lagged associations with EMA predicting compulsivity at the next time point.	28](#_Toc230285100)

[Table S16: Differences between participants in the OCD behaviors group with and without a diagnosis in time-lagged associations with compulsivity predicting EMA at the next time point.	29](#_Toc230285101)

[MATERIALS AND METHODS	30](#_Toc230285102)

[Meta Mind (Perceptual decision-making task) – Metacognitive confidence bias	31](#_Toc230285103)

[Cannon Blast (Two-Step Task) – Model-based planning	34](#_Toc230285104)

[Digital questionnaire response time – Cognitive processing speed	37](#_Toc230285105)

[REFERENCES	38](#_Toc230285106)

# **RESULTS**

## **Figure S1: Main effects per group and group comparisons for the contemporaneous associations between EMA and compulsivity.**

Point estimates show the main effects for each behavior type and each EMA item. Asterisks on the right side of each row depict significant differences between the behavior types.

Annotation: * = p < .05; ** = p < .01; *** = p < .001

## **Table S1: Differences between the compulsive behavior types in contemporaneous associations between EMA and compulsivity (comp.).**

Contemporaneous associations were estimated using the residuals extracted from the two directional models and the resulting regression parameters were averaged across both directions. Accordingly, differences between the compulsive behavior types were considered significant if both directional p_FDR_ < 0.05.

| Comparison | Experience | *mean* Δβ | *p_FDR_*  EMA→Comp. | *p_FDR_*  Comp.→EMA |
| --- | --- | --- | --- | --- |
| Phone checking - Binge eating | Urge | 0.12 | .917 | < .001 |
| Phone checking - OCD behaviors | Urge | -0.06 | < .001 | .099 |
| **Binge eating - OCD behaviors** | **Urge** | **-0.18** | **< .001***** | **.048*** |
| Phone checking - Binge eating | Try to resist | 0.09 | .881 | .135 |
| Phone checking - OCD behaviors | Try to resist | -0.02 | .540 | .357 |
| Binge eating - OCD behaviors | Try to resist | -0.11 | .472 | .314 |
| Phone checking - Binge eating | Feel in control | 0.00 | .616 | .296 |
| Phone checking - OCD behaviors | Feel in control | -0.12 | .752 | .011 |
| Binge eating - OCD behaviors | Feel in control | -0.13 | .308 | .116 |
| Phone checking - Binge eating | Obsessive thoughts | 0.05 | .863 | .263 |
| Phone checking - OCD behaviors | Obsessive thoughts | -0.19 | < .001 | .099 |
| **Binge eating - OCD behaviors** | **Obsessive thoughts** | **-0.25** | **< .001***** | **.004**** |
| Phone checking - Binge eating | Stressful events | 0.02 | .863 | .694 |
| **Phone checking - OCD behaviors** | **Stressful events** | **-0.14** | **< .001***** | **.049*** |
| **Binge eating - OCD behaviors** | **Stressful events** | **-0.16** | **< .001***** | **.031*** |
| **Phone checking - Binge eating** | **Bored** | **0.21** | **.001**** | **< .001***** |
| **Phone checking - OCD behaviors** | **Bored** | **0.27** | **< .001***** | **< .001***** |
| Binge eating - OCD behaviors | Bored | 0.06 | .413 | .201 |
| Phone checking - Binge eating | Guilty | 0.01 | .681 | .415 |
| Phone checking - OCD behaviors | Guilty | 0.01 | .377 | .357 |
| Binge eating - OCD behaviors | Guilty | -0.00 | .683 | .778 |
| Phone checking - Binge eating | Happy | -0.10 | .481 | .070 |
| Phone checking - OCD behaviors | Happy | -0.04 | .752 | .202 |
| Binge eating - OCD behaviors | Happy | 0.06 | .242 | .349 |
| Phone checking - Binge eating | Tired | 0.02 | .863 | .563 |
| Phone checking - OCD behaviors | Tired | 0.05 | .752 | .202 |
| Binge eating - OCD behaviors | Tired | 0.03 | .683 | .385 |
| Phone checking - Binge eating | Anxious | 0.06 | .681 | .263 |
| Phone checking - OCD behaviors | Anxious | -0.11 | < .001 | .202 |
| **Binge eating - OCD behaviors** | **Anxious** | **-0.17** | **< .001***** | **.031*** |
| *Note.* * p < .05, ** p < .01, *** p < .001 | | | | |

## **Figure S2: Main effects per group and group comparisons for time-lagged associations with EMA predicting compulsivity at the next time point.**

Point estimates and standard errors show the main effects for each behavior type and each EMA item. Asterisks on the right side of each row depict significant differences between the behavior types.

Annotation: * = p < .05; ** = p < .01; *** = p < .001

## **Table S2: Differences between the compulsive behavior types in time-lagged associations with EMA predicting compulsivity at the next time point.**

| Comparison | Experience | Δβ | *SE* | *t* | *p_FDR_* |
| --- | --- | --- | --- | --- | --- |
| Phone checking - Binge eating | Urge | 0.06 | 0.04 | 1.53 | .392 |
| Phone checking - OCD behaviors | Urge | 0.06 | 0.04 | 1.51 | .573 |
| Binge eating - OCD behaviors | Urge | -0.00 | 0.04 | -0.01 | .995 |
| Phone checking - Binge eating | Try to resist | 0.05 | 0.04 | 1.31 | .409 |
| Phone checking - OCD behaviors | Try to resist | 0.05 | 0.04 | 1.50 | .573 |
| Binge eating - OCD behaviors | Try to resist | 0.01 | 0.03 | 0.18 | .964 |
| Phone checking - Binge eating | Feel in control | -0.05 | 0.04 | -1.45 | .392 |
| Phone checking - OCD behaviors | Feel in control | -0.08 | 0.03 | -2.30 | .573 |
| Binge eating - OCD behaviors | Feel in control | -0.03 | 0.03 | -0.83 | .817 |
| Phone checking - Binge eating | Obsessive thoughts | 0.04 | 0.04 | 0.97 | .790 |
| Phone checking - OCD behaviors | Obsessive thoughts | 0.03 | 0.04 | 0.65 | .903 |
| Binge eating - OCD behaviors | Obsessive thoughts | -0.02 | 0.04 | -0.48 | .964 |
| Phone checking - Binge eating | Stressful events | 0.07 | 0.03 | 2.20 | .392 |
| Phone checking - OCD behaviors | Stressful events | 0.02 | 0.03 | 0.80 | .704 |
| Binge eating - OCD behaviors | Stressful events | -0.05 | 0.03 | -1.61 | .406 |
| Phone checking - Binge eating | Bored | -0.02 | 0.03 | -0.52 | .868 |
| Phone checking - OCD behaviors | Bored | 0.01 | 0.03 | 0.17 | .964 |
| Binge eating - OCD behaviors | Bored | 0.02 | 0.03 | 0.76 | .964 |
| Phone checking - Binge eating | Guilty | 0.03 | 0.04 | 0.74 | .844 |
| Phone checking - OCD behaviors | Guilty | 0.02 | 0.04 | 0.53 | .949 |
| Binge eating - OCD behaviors | Guilty | -0.01 | 0.04 | -0.31 | .964 |
| Phone checking - Binge eating | Happy | -0.05 | 0.03 | -1.56 | .392 |
| Phone checking - OCD behaviors | Happy | 0.03 | 0.03 | 1.16 | .573 |
| Binge eating - OCD behaviors | Happy | 0.08 | 0.03 | 2.92 | .089 |
| Phone checking - Binge eating | Tired | 0.05 | 0.03 | 1.85 | .392 |
| Phone checking - OCD behaviors | Tired | 0.00 | 0.02 | 0.03 | .979 |
| Binge eating - OCD behaviors | Tired | -0.05 | 0.02 | -2.06 | .322 |
| Phone checking - Binge eating | Anxious | 0.04 | 0.03 | 1.19 | .392 |
| Phone checking - OCD behaviors | Anxious | -0.04 | 0.03 | -1.40 | .573 |
| Binge eating - OCD behaviors | Anxious | -0.08 | 0.03 | -2.75 | .089 |
| *Note.* * p < .05, ** p < .01, *** p < .001 | | | | | |

## **Figure S3: Main effects per group and group comparisons for the autocorrelation of EMA.**

Point estimates and standard errors show the main effects for each behavior type and each EMA item. Asterisks on the right side of each row depict significant differences between the behavior types.

Annotation: * = p < .05; ** = p < .01; *** = p < .001

## **Table S3: Differences between the compulsive behavior types in autocorrelation of EMA.**

| Comparison | Experience | Δβ | *SE* | *t* | *p_FDR_* |
| --- | --- | --- | --- | --- | --- |
| **Phone checking - Binge eating** | **Time spent** | **0.17** | **0.04** | **4.08** | **.001**** |
| Phone checking - OCD behaviors | Time spent | 0.04 | 0.04 | 0.99 | .593 |
| **Binge eating - OCD behaviors** | **Time spent** | **-0.13** | **0.04** | **-3.12** | **.015*** |
| Phone checking - Binge eating | Urge | 0.12 | 0.04 | 2.95 | .105 |
| Phone checking - OCD behaviors | Urge | 0.09 | 0.04 | 2.35 | .314 |
| Binge eating -  OCD behaviors | Urge | -0.03 | 0.04 | -0.71 | .733 |
| Phone checking - Binge eating | Try to resist | 0.13 | 0.05 | 2.89 | .127 |
| Phone checking - OCD behaviors | Try to resist | 0.09 | 0.04 | 2.17 | .494 |
| Binge eating -  OCD behaviors | Try to resist | -0.04 | 0.04 | -0.86 | .560 |
| Phone checking - Binge eating | Feel in control | 0.07 | 0.05 | 1.55 | .949 |
| Phone checking - OCD behaviors | Feel in control | 0.03 | 0.04 | 0.68 | .949 |
| Binge eating -  OCD behaviors | Feel in control | -0.04 | 0.04 | -0.97 | .949 |
| Phone checking - Binge eating | Obsessive thoughts | 0.05 | 0.05 | 0.90 | .940 |
| Phone checking - OCD behaviors | Obsessive thoughts | 0.06 | 0.05 | 1.28 | .940 |
| Binge eating -  OCD behaviors | Obsessive thoughts | 0.01 | 0.04 | 0.31 | .940 |
| Phone checking - Binge eating | Stressful events | 0.03 | 0.04 | 0.80 | .727 |
| Phone checking - OCD behaviors | Stressful events | -0.03 | 0.04 | -0.75 | .714 |
| Binge eating -  OCD behaviors | Stressful events | -0.06 | 0.04 | -1.59 | .523 |
| Phone checking - Binge eating | Bored | -0.11 | 0.04 | -2.70 | .228 |
| Phone checking - OCD behaviors | Bored | -0.05 | 0.04 | -1.34 | .492 |
| Binge eating -  OCD behaviors | Bored | 0.06 | 0.04 | 1.56 | .894 |
| Phone checking - Binge eating | Guilty | 0.09 | 0.05 | 1.85 | .800 |
| Phone checking - OCD behaviors | Guilty | 0.04 | 0.05 | 0.89 | .819 |
| Binge eating -  OCD behaviors | Guilty | -0.05 | 0.05 | -1.15 | .728 |
| Phone checking - Binge eating | Happy | 0.04 | 0.04 | 1.01 | .905 |
| Phone checking - OCD behaviors | Happy | 0.02 | 0.04 | 0.54 | .961 |
| Binge eating -  OCD behaviors | Happy | -0.02 | 0.04 | -0.55 | .905 |
| Phone checking - Binge eating | Tired | -0.02 | 0.04 | -0.49 | .990 |
| Phone checking - OCD behaviors | Tired | 0.01 | 0.04 | 0.18 | .990 |
| Binge eating -  OCD behaviors | Tired | 0.03 | 0.04 | 0.70 | 1.00 |
| Phone checking - Binge eating | Anxious | 0.09 | 0.04 | 2.26 | .198 |
| Phone checking - OCD behaviors | Anxious | 0.10 | 0.04 | 2.79 | .172 |
| Binge eating -  OCD behaviors | Anxious | 0.01 | 0.04 | 0.28 | .982 |
| *Note.* * p < .05, ** p < .01, *** p < .001 | | | | | |

## **Figure S4: Main effects per group and group comparisons for contemporaneous associations between EMA and cognitive processing speed as measured by DQRT.**

Point estimates show the main effects for each behavior type and each EMA item. Asterisks on the right side of each row depict significant differences between the behavior types.

Annotation: * = p < .05; ** = p < .01; *** = p < .001

## **Table S4: Differences between the compulsive behavior types in contemporaneous associations between EMA and cognitive processing speed as measured by DQRT.**

Contemporaneous associations were estimated using the residuals extracted from the two directional models and the resulting regression parameters were averaged across both directions. Accordingly, differences between the compulsive behavior types were considered significant if both directional p_FDR_ < 0.05.

| Comparison | Experience | *mean* Δβ | *p_FDR_*  EMA→DQRT | *p_FDR_*  DQRT→EMA |
| --- | --- | --- | --- | --- |
| Phone checking - Binge eating | Compulsivity | -0.00 | .949 | .967 |
| Phone checking - OCD behaviors | Compulsivity | 0.00 | .868 | .916 |
| Binge eating - OCD behaviors | Compulsivity | 0.00 | .907 | .891 |
| Phone checking - Binge eating | Urge | 0.02 | .949 | .967 |
| Phone checking - OCD behaviors | Urge | 0.01 | .868 | .916 |
| Binge eating - OCD behaviors | Urge | -0.01 | .907 | .891 |
| Phone checking - Binge eating | Try to resist | 0.01 | .949 | .967 |
| Phone checking - OCD behaviors | Try to resist | 0.00 | .974 | .916 |
| Binge eating - OCD behaviors | Try to resist | -0.00 | .907 | .891 |
| Phone checking - Binge eating | Feel in control | 0.01 | .949 | .967 |
| Phone checking - OCD behaviors | Feel in control | 0.01 | .974 | .916 |
| Binge eating - OCD behaviors | Feel in control | 0.00 | .907 | .891 |
| Phone checking - Binge eating | Obsessive thoughts | 0.01 | .949 | .967 |
| Phone checking - OCD behaviors | Obsessive thoughts | 0.03 | .974 | .916 |
| Binge eating - OCD behaviors | Obsessive thoughts | 0.02 | .907 | .891 |
| Phone checking - Binge eating | Stressful events | -0.01 | .949 | .967 |
| Phone checking - OCD behaviors | Stressful events | 0.04 | .428 | .916 |
| Binge eating - OCD behaviors | Stressful events | 0.05 | .731 | .891 |
| Phone checking - Binge eating | Bored | 0.01 | .949 | .967 |
| Phone checking - OCD behaviors | Bored | 0.08 | .108 | .344 |
| Binge eating - OCD behaviors | Bored | 0.07 | .180 | .344 |
| Phone checking - Binge eating | Guilty | 0.00 | .949 | .967 |
| Phone checking - OCD behaviors | Guilty | 0.02 | .868 | .916 |
| Binge eating - OCD behaviors | Guilty | 0.02 | .907 | .891 |
| Phone checking - Binge eating | Happy | -0.04 | .949 | .967 |
| Phone checking - OCD behaviors | Happy | -0.04 | .868 | .916 |
| Binge eating - OCD behaviors | Happy | -0.00 | .907 | .891 |
| Phone checking - Binge eating | Tired | -0.00 | .949 | .967 |
| Phone checking - OCD behaviors | Tired | -0.03 | .974 | .916 |
| Binge eating - OCD behaviors | Tired | -0.03 | .907 | .891 |
| Phone checking - Binge eating | Anxious | -0.03 | .949 | .967 |
| Phone checking - OCD behaviors | Anxious | -0.01 | .974 | .916 |
| Binge eating - OCD behaviors | Anxious | 0.02 | .907 | .891 |
| *Note.* * p < .05, ** p < .01, *** p < .001 | | | | |

## **Comparisons between individuals with a diagnosis and those without in the OCD behaviors and binge eating groups**

Participants in the OCD behaviors group and binge-eating group were a combination of citizen scientists and those with confirmed diagnoses. In the OCD behaviors group, 39/63 (62%) had a confirmed diagnosis and in the binge eating group, 17/53 (32%) had a confirmed diagnosis. We confirmed that individuals with a diagnosis reported more time spent performing the compulsion (Δβ=0.87, *p*_FDR_=0.008; Fig. S8A). We further found they endorsed higher levels of obsessive thoughts (Δβ=1.24, *p*_FDR_<0.001), stress (Δβ=0.84, *p*_FDR_=0.008), boredom (Δβ=0.88, *p*_FDR_=0.008), guilt (Δβ=0.68, *p*_FDR_=0.045), and anxiety (Δβ=0.69, *p*_FDR_=0.045), and lower levels of control (Δβ=-0.84, *p*_FDR_=0.03), while we found no differences for urges, attempts to resist, and feeling happy or tired (0.19<absolute β<0.56, all *p*_FDR_>0.05). We found no difference in variance in symptom levels in any of the EMA items for participants with a diagnosis compared to those without (all *p*_FDR_>0.05; Fig. S8B). Individuals with a diagnosis had higher scores for CIT (Δβ=0.71, p_FDR_<0.001; Fig. S8C) compared to citizen scientists but we did not find a difference between the groups for AD (Δβ=0.33, *p*_FDR_>0.05). For cross-sectional assessments of cognition (Fig. S8D), we found no differences between the groups for cognitive processing speed as measured by mean DQRT (Δβ=0.33), metacognitive confidence bias (Δβ=-0.19), or model-based planning (Δβ=-0.11).

## **Figure S8: Across the OCD behaviors and binge eating groups, experiences associated with compulsions are more pronounced in individuals with a diagnosis.**

(A) Mean symptom levels measured across 11 ecological momentary assessment items are higher for compulsivity, obsessive thoughts, stress, boredom, guilt and anxiety, and lower for feeling in control in participants with a diagnosis compared to those without. (B) Variance in symptom levels does not differ between participants with a diagnosis compared to participants without a diagnosis for any of the 11 ecological momentary assessment items. (C) For transdiagnostic psychopathology, participants with a diagnosis reported higher compulsivity and intrusive thoughts but did not differ from participants without a diagnosis on their anxious-depression. (D) One-time assessments of cognitive processing speed (Digital Questionnaire Response Time (DQRT)), mean metacognitive confidence, and model-based planning show no group-level differences.
In all panels, coloured dots show individual-level data averaged across all available assessments within person while boxplots summarize the data across participants with the box outlines depicting the interquartile range and the line in the middle depicting the median.

Annotation: * = p < .05; ** = p < .01; *** = p < .001

## **Assessing signal contained in the behavioral time-series data for autocorrelation analyses**

To assess whether the sampling frequency for our ecological momentary assessments (EMA) resulted in sufficient signal given the lag of five hours between assessments, we randomly redistributed the assessment timepoints within person, thus eliminating the temporal order inherent to the data, and reran our autocorrelation models on this permuted time series. We repeated this procedure 1000 times to obtain a distribution of coefficients for the autocorrelation of each of the 11 EMA items, reflecting the assumption of a fully random sampling of datapoints. We then summed the number of cases where the autocorrelation coefficients calculated from permuted data were higher or equal to the autocorrelation coefficients calculated from the original data, and divided this number by 1000 (the number of permutations) to obtain a p-value.

For the full set of 11 EMA items, none of the autocorrelation coefficients calculated from permuted time series reached or exceeded the coefficients calculated from the original time series (Figure S9). This provides strong evidence that given the sampling frequency, the EMA time series do not reflect randomly selected datapoints but instead contain a meaningful temporal order to calculate autocorrelation from.

## **Figure S9: Ecological momentary assessment (EMA) time series with a five-hour lag between assessments contain sufficient information to calculate autocorrelation.**

To confirm that all eleven EMA time series contained a meaningful temporal order to calculate autocorrelation from, we randomly redistributed the assessment time points 1000 times, each time re-running the autocorrelation model to to obtain a distribution of coefficients for the autocorrelation of each of the eleven EMA items. For each item, the distribution shows the autocorrelation coefficients calculated from the 1000 runs of permuted assessments. The vertical line in each plot shows the real autocorrelation coefficient as calculated from the original, unaltered time series. None of the autocorrelation coefficients calculated from permuted data were higher or equal to the autocorrelation coefficients calculated from the original data, indicating that all EMA time series contain a meaningful temporal order to calculate autocorrelation from.

Annotation: * = p < .05; ** = p < .01; *** = p < .001

**Table S10: Demographics for non-completers of the Pattern Wise module who were not included in the main analyses, split by compulsive behavior type and diagnostic status.**

We compared mean age, gender distribution, and educational attainment between the three compulsive behavior types, averaging across individuals with and without a diagnosis within one behavior type.

|  |  | **OCD behaviours** | | **Binge eating** | | **Phone checking** |
| --- | --- | --- | --- | --- | --- | --- |
|  |  | Diagnosed  N=2 | No diagnosis  N=195 | Diagnosed  N=2 | No diagnosis  N=269 | N=738 |
| **Demographics** | | | | | | |
| Age (*p* < .001***) | | 28.0±2.8 | 53.4±12.5 | 24.5±2.1 | 48.1±12.9 | 49.2±14.8 |
| Gender (*p* = .004**) | | 2 female | 139 female  54 male  2 non-cisgender | 2 female | 228 female  37 male  4 non-cisgender | 546 female  170 male  20 non-cisgender  2 prefer not to answer |
| Education (*p* < .001***) | | 3.0 | 2.6 | 3.5 | 3.0 | 2.9 |

**Figure S11: Response distributions for each of the 11 ecological momentary assessment variables by compulsive behavior types.**

Participants responded using an 8-point Likert response scale, which numerically ranged from zero to seven (shown on the x-axis). The first 8 items (Compulsivity to Guilty) are unipolar with the response value ‘0’ corresponding to ‘Not true’ and the response value ‘7’ corresponding to ‘Extremely true’. The remaining 3 items are bipolar and ask for participants’ affect on a continuum from sad to happy (Happy), energetic to tired (Tired), and relaxed to anxious (Anxious).

## **Sensitivity analysis addressing zero-inflation in the compulsivity outcome through a hurdle model**

To assess whether directly accounting for the zero-inflation in our outcome measure, compulsivity, leads to different results compared to the multilevel vector autoregression models we report in the main text, we ran a hurdle model. Hurdle models assume that zeros are the consequence of not crossing the threshold (or hurdle) of experiencing the outcome, consistent with individuals in our sample who, generally, could experience compulsions before or during any of the assessment time points, but for many of those occasions would not perform compulsions, thus, reporting a zero. Hurdle models reflect a two-step process wherein first, a logistic regression model is fit to assess the probability of observing a zero versus observing any non-zero value (crossing the hurdle). Then, a gamma regression is fit to all non-zero values where the hurdle has been crossed (conditional model). We used the glmmTMB package (1) in R to fit a hurdle model using a zero-inflated gamma distribution to account for the right-skew in non-zero values of the compulsivity distribution, and a log link function to ensure predicted values are positive. We used the same regression formula for predicting compulsivity from the preceding assessment time point of each other EMA variable as described in the methods section for the multilevel vector autoregressive models (mlVAR) for both the zero-inflation model (logistic regression) and the conditional model (gamma regression). All predictor variables remained the same. Only for compulsivity as our dependent variable, we now used the raw scores instead of scaled values to preserve the meaning of zeros. To be able to compare the results of the hurdle models to the mlVAR models described in the main text, we report t-values and p-values corrected for multiple comparisons by controlling the false discovery rate (FDR) using the Benjamini-Hochberg method (2). by controlling the false discovery rate (FDR) using the Benjamini-Hochberg method (2). We find that the zero-inflation component of the hurdle model (HM) leads to results that are highly comparable to the mlVAR model we report as our main analytical approach. For both models, only urges (mlVAR: *t*=4.84, *p_FDR_*<.001; HM: *t*=-4.06, *p_FDR_*<.001) and trying to resist the compulsion (mlVAR: *t*=2.94, *p_FDR_*=.02; HM: *t*=-3.49, *p_FDR_*=.002) predicted compulsivity at the next assessment time point (Table S12). Note that t-values originating from the zero-inflation component of the HM will show an inverted sign as the zero-inflation component predicts the probability of observing a zero. These findings indicate that both models, although reflecting different assumptions, lead to highly comparable results.

**Table S12: Comparison between the outcomes of a multilevel vector autoregressive model (mlVAR) reported in the main text and a hurdle model which addresses zero-inflation in our main outcome variable ‘compulsivity’.**

We ran models predicting compulsivity from the preceding assessment time point of each other EMA variable (column ‘Experience’ in the table). Results listed in the mlVAR column are taken from the analyses reported in the main text. For the hurdle model, we report the zero-inflation component (logistic regression model predicting the probability of observing a zero) and the conditional component (gamma regression model predicting all non-zero values for compulsivity). Significant outcomes are shaded in gray.

|  | mlVAR | | Hurdle model | | | |
| --- | --- | --- | --- | --- | --- | --- |
|  |  | | Zero-inflation component | | Conditional component | |
| Experience | *t* | *p_FDR_*  EMA→Comp. | *t* | *p_FDR_*  EMA→Comp. | *t* | *p_FDR_*  EMA→Comp. |
| Urge | **4.84** | **< .001***** | **-4.06** | **< .001***** | **3.07** | **.021*** |
| Try to resist | **2.94** | **.019*** | **-3.49** | **.002**** | 1.28 | .403 |
| Feel in control | -1.38 | .343 | 0.93 | .599 | -0.26 | .886 |
| Obsessive thoughts | 0.42 | .841 | -0.32 | .939 | 0.41 | .886 |
| Stressful events | 1.96 | .173 | -0.01 | .988 | 2.54 | .055 |
| Bored | 1.09 | .464 | -1.22 | .599 | 0.35 | .886 |
| Guilty | 1.76 | .202 | -0.96 | .599 | 2.29 | .073 |
| Happy | 0.11 | .914 | -0.16 | .968 | -0.12 | .908 |
| Tired | -0.12 | .914 | -0.81 | .599 | 0.62 | .886 |
| Anxious | 0.63 | .757 | 0.87 | .599 | 1.61 | .269 |

**Table S13: Compulsive behaviors participants selected to track in the neureka app and other compulsions patients reported to experience but did not track in the app.**

In the Pattern Wise module of the neureka app, all participants were required to select one compulsive behavior they wanted to track for the 6 weeks of the study which are displayed in the section ‘Tracked compulsions’. For patients with a diagnosis, we collected all current compulsions which are displayed in the section ‘Additional compulsions experienced by participants, but not necessarily tracked’, and confirmed their main compulsive behavior during the screening interview. If patients felt that the pre-specified compulsions in the neureka app did not fit them, they were instructed to select the ‘Other’ option and focus on their main compulsion. The compulsions patients tracked who selected the ‘Other’ option are displayed under ‘Tracked compulsions for participants who selected ‘Other’. For citizen scientists, in-depth information on compulsions were not available. Hence, participants selecting the ‘Other’ category were not included in the sample.

|  |  | **OCD behaviours** | | **Binge eating** | | **Phone checking** |
| --- | --- | --- | --- | --- | --- | --- |
|  |  | Diagnosed  N=39 | No diagnosis  N=24 | Diagnosed  N=17 | No diagnosis  N=35 | N=64 |
| **Tracked compulsions** | | | | | | |
| Checking locks | | 3 | 18 |  |  |  |
| Checking appliances | | 4 | 2 |  |  |  |
| Cleaning | | 10 | 3 |  |  |  |
| Counting | | 1 | 1 |  |  |  |
| Repeating | | 2 |  |  |  |  |
| Eating | |  |  | 17 | 17 |  |
| Checking your phone | |  |  |  |  | 64 |
| **Tracked compulsions for participants who selected ‘Other’** | | | | | | |
| Health-related checking | | 1 |  |  |  |  |
| Mental rumination | | 7 |  |  |  |  |
| Throat clearing | | 2 |  |  |  |  |
| Rearranging | | 1 |  |  |  |  |
| Washing | | 2 |  |  |  |  |
| Location-related checking | | 1 |  |  |  |  |
| Reassurance seeking | | 1 |  |  |  |  |
| Skin-picking | | 1 |  |  |  |  |
| Safe/”just right” rituals (counting numbers, tapping etc.) | | 3 |  |  |  |  |
| **Additional compulsions experienced by participants, but not necessarily tracked** | | | | | | |
| Checking locks | | 5 |  |  |  |  |
| Checking appliances | | 9 |  |  |  |  |
| Health-related checking | | 4 |  |  |  |  |
| Location-related checking | | 1 |  |  |  |  |
| Reassurance seeking | | 1 |  |  |  |  |
| Repeating | | 2 |  |  |  |  |
| Cleaning/washing | | 21 |  |  |  |  |
| Safe/”just right” rituals (counting numbers, tapping etc.) | | 11 |  | 1 |  |  |
| Mental rumination | | 11 |  |  |  |  |
| Ordering/rearranging | | 6 |  |  |  |  |
| Throat clearing | | 2 |  |  |  |  |
| Skin-picking | | 2 |  |  |  |  |
| Hair-pulling | | 1 |  | 1 |  |  |

## **Table S14: Differences between participants in the OCD behaviors group with and without a diagnosis in contemporaneous associations between EMA and compulsivity (comp.).**

Contemporaneous associations were estimated using the residuals extracted from the two directional models and the resulting regression parameters were averaged across both directions. Accordingly, differences between participants with and without a diagnosis were considered significant if both directional p_FDR_ < 0.05.

**Comparison: Participants without a diagnosis – participants with a diagnosis**

| Experience | *mean* Δβ | *p_FDR_*  EMA→Comp. | *p_FDR_*  Comp.→EMA |
| --- | --- | --- | --- |
| Urge | -0.14 | .09 | .44 |
| Try to resist | 0.06 | .74 | .099 |
| Feel in control | 0.18 | .41 | .05 |
| **Obsessive thoughts** | **-0.32** | **<.001***** | **.02*** |
| Stressful events | -0.19 | .01 | .13 |
| Bored | -0.04 | .63 | .61 |
| Guilty | -0.08 | .01 | .76 |
| Happy | 0.17 | .09 | .004 |
| Tired | -0.10 | .21 | .13 |
| **Anxious** | **-0.28** | **<.001***** | **<.001***** |

## **Table S15: Differences between participants in the OCD behaviors group with and without a diagnosis in time-lagged associations with EMA predicting compulsivity at the next time point.**

**Comparison: Participants without a diagnosis – participants with a diagnosis**

| Experience | Δβ | *SE* | *t* | *p_FDR_* |
| --- | --- | --- | --- | --- |
| Urge | 0.05 | 0.06 | 0.77 | .628 |
| Try to resist | 0.11 | 0.06 | 1.85 | .446 |
| Feel in control | -0.03 | 0.06 | -0.59 | .692 |
| Obsessive thoughts | -0.03 | 0.07 | -0.49 | .697 |
| Stressful events | -0.05 | 0.06 | -0.97 | .549 |
| Bored | 0.07 | 0.06 | 1.23 | .549 |
| Guilty | 0.09 | 0.08 | 1.16 | .549 |
| Happy | -0.02 | 0.05 | -0.33 | .742 |
| Tired | 0.07 | 0.04 | 1.70 | .446 |
| Anxious | -0.05 | 0.05 | -1.02 | .549 |

## **Table S16: Differences between participants in the OCD behaviors group with and without a diagnosis in time-lagged associations with compulsivity predicting EMA at the next time point.**

**Comparison: Participants without a diagnosis – participants with a diagnosis**

| Experience | Δβ | *SE* | *t* | *p_FDR_* |
| --- | --- | --- | --- | --- |
| Urge | -0.14 | 0.09 | -1.62 | .933 |
| Try to resist | 0.01 | 0.06 | 0.84 | .933 |
| Feel in control | 0.03 | 0.06 | 0.45 | .933 |
| Obsessive thoughts | -0.01 | 0.05 | -0.22 | .933 |
| Stressful events | 0.05 | 0.07 | 0.64 | .933 |
| Bored | 0.03 | 0.07 | 0.36 | .933 |
| Guilty | -0.02 | 0.05 | -0.37 | .933 |
| Happy | -0.03 | 0.05 | -0.56 | .933 |
| Tired | -0.001 | 0.06 | -0.02 | .984 |
| Anxious | -0.04 | 0.05 | -0.77 | .933 |

# **MATERIALS AND METHODS**

**Figure S5. Flowchart showing the allocation of participants to the three compulsive behavior groups.**

We recruited individuals with OCD compulsions or eating compulsions in collaboration with Hertfordshire Partnership NHS Foundation Trust (Hertfordshire), South West London and St George's Mental Health NHS Trust (SW London and St George’s), and the Icahn School of Medicine at Mount Sinai (Mount Sinai). Additionally, we recruited patients from an existing database as they previously participated in research on compulsive behaviors and had consented to being contacted for future studies (Existing Database). All patients completed a screening interview using the Mini-International Neuropsychiatric Interview(3) and were only included if they fulfilled the criteria for current obsessive-compulsive disorder, binge-eating disorder or bulimia nervosa. Additionally, we included citizen scientists who were subscribed to ‘Pattern Wise’, a digital tracker for compulsivity in the research-focused smartphone app ‘Neureka’, and chose to track one of either traditional OCD behaviors (this included checking locks, cleaning, checking appliances, and counting), eating, or phone-checking.

## **Meta Mind (Perceptual decision-making task) – Metacognitive confidence bias**

Meta Mind is a gamified version of a perceptual decision-making task(4) which allows to estimate a participant’s metacognitive bias and metacognitive efficiency. In perceptual decision-making tasks, participants are asked to make repeated difficult decisions between two options, often which of two shapes containing a large number of dots contains more dots than the other, before being asked to rate their confidence in their last choice being correct. Metacognitive bias then refers to an individual’s tendency to rate their confidence higher or lower on average, while metacognitive efficiency reflects the extent to which individual confidence ratings match accuracy, that is if a high confidence is given only in those trials were the correct option was indeed selected. The current work only used metacognitive bias or mean confidence ratings as measure of interest as previous work has shown that difference in metacognitive bias are linked to individuals score on a spectrum of transdiagnostic mental health symptoms, including anxious-depression and compulsivity and intrusive thoughts(5).

In Meta Mind, players steer a spaceship through the brain and make choices based on the series of stimuli they pass along the way. On each trial, players are presented with two (identical) moving shapes where one shape contains 100 dots and the other shape will contain a number of dots that varies between 101 and 149. The size of the difference in dots between the two shapes determines the difficulty of the trial with change in dot difference being calculated in a log-space. Players are instructed to move their spaceship to the shape they believe contains more dots by tapping the side of the screen the shape of their choice is displayed on. Next, they rate their confidence on a six-point scale ranging from low to high. After a set of 20 trials, players also evaluate their overall (global) performance based on how they think they performed across the full 20 trials. However, this global confidence estimate was not used in the current work.

Meta Mind shows three instructed tutorial trials followed by five sets of 20 game trials. The first 20 trials are not used for analyses as they are used to burn in the staircase algorithm. Meta Mind uses a ‘two-down one-up’ staircase procedure, in which the task becomes easier (i.e. a larger dot difference between shapes) after each incorrect response and more difficult (i.e. a smaller dot difference between shapes) after two consecutive correct responses. This maintains objective performance as reflected by each player’s average accuracy between 0.6 and 0.85, which ensured that estimates of confidence were not confounded by performance differences. While the tutorial trials all start with the lowest difficulty (dot difference of 49), the first game trial starts with a difficulty level of 13 (dot difference of 15 and a corresponding log value of 2.7), which adapts based on the staircasing over the subsequent trials.

A prior paper validated this task in detail(5). In brief, there was a moderate positive association between mean confidence derived from Meta Mind and a traditional version of a perceptual decision-making task (*r*=0.64, *p*<0.001). The split-half reliability for mean confidence was excellent for both the traditional task (*r*=0.98, *p*<0.001) and Meta Mind (*r*=0.91, *p*<0.001). The test-retest reliability for mean confidence as measured using Meta Mind and an intraclass correlation (ICC; two-way mixed-effects model with absolute agreement, single rater) analysis was good with *ICC*=0.86 (*p*<0.001) assessed over a variable interval (median 2 days, SD=7.74 days).

**Figure S6. Task structure of Meta Mind, a smartphone game to assess metacognitive confidence bias.** In Meta Mind, players are instructed to navigate their ship to the stimulus containing more dots. At the start of each Meta Mind round (N = 20 trials), players are shown a screen with no stimuli for 1000 ms. Stimuli then appear at the top of the screen and drift towards the bottom. Stimuli contain dots for 300 ms. After the dots disappear, blank shapes appear on screen for up to 2000 ms, until a choice is made. Players tap the left or right side of the screen to choose the stimulus they think contained more dots Missed trials are recorded and repeated with shapes that have the same dot difference. After Meta Mind players choose a side, they then rate their local confidence in the accuracy of that choice on the trial, with unlimited time. After 20 Meta Mind trials, players evaluate their overall accuracy on that block (round-level, global confidence).

Figure and legend reproduced with permission from Fox, McDonogh (5) published in Scientific Reports under the CC-BY-4.0 license (https://creativecommons.org/licenses/by/4.0/).

## **Cannon Blast (Two-Step Task) – Model-based planning**

Cannon Blast is a gamified version of the two-step reinforcement learning task(6) which estimates a participant’s ‘model-based’ tendency, representing the extent to which they make decisions using a mental map of action-outcome associations. In Cannon Blast, participants have 100 shots from a cannon to hit and collect as many diamonds as possible. To supply the cannon with balls to shoot, participants select from one of two containers with the left container having more purple-colored balls (80%) and the right container having more pink-colored balls (80%). Balls drawn from a container would either be good balls which could be used to collect the diamond (reward trial) or disintegrate upon firing, making it impossible to collect the diamond (unrewarded trial). However, even if the participant received a good ball, collecting the diamond was still dependent on their aim and timing. The probability that pink or purple balls are more likely to be good drifted over the course of the game and, thus, had to be constantly tracked by participants to increase their chance of collecting diamonds. In contrast to the original two-step task, Cannon Blast only presents participants with one stage of making a choice (which container to draw balls from). This was done to increase the importance of model-based contributions in this first-stage choice(7).

Paid participants were instructed to first complete a passive tutorial that explains the task and the goal to collect as many diamonds as possible. Then, they completed 100 trials on an easy difficulty level with a static or slowly moving diamond, or static barriers that limited the participants aim. As the citizen scientists were free to explore the Neureka app, they did not receive specific instructions but we included any valid set of 100 trials of Cannon Blast they had completed on the easy difficulty level. The outcome of interest for Cannon Blast is the model-based index (MBI) which is calculated using a hierarchical logistic regression model, predicting the binary choice between the two containers as a function of reward (good or bad ball) and transition (which color was shot from the chosen container). The model-based index reflects in how far participants use their knowledge about previous transitions and rewards to make a subsequent choice.

A prior paper validated this task in detail(7). In brief, there was a moderate positive association between MBI derived from Cannon Blast and the traditional two-step reinforcement learning task (*r*=0.40, *p*=.002). Split-half reliability for MBI were high for both the traditional task (*r* [*CI*]=0.81 [0.70-0.88], *p*<.001) and Cannon Blast (*r* [*CI*]=0.78 [0.66-0.87], *p*<.001). The test-retest reliability was *r* (423)=0.63 assessed over a variable interval (median 4 days).

**Figure S7. Task structure of Cannon Blast, a smartphone game to assess model-based planning.** A. In this game, participants’ goal is to shoot as many diamonds as possible before their total number of shots (100 per block) runs out. To do so, they must aim a central cannon and then select which circular container to draw from. B. Purple and pink balls dynamically bounce around each of the flanked containers which depict the probability of a pink or purple ball being released. For example, the left container displays 8 purple balls and releases a purple ball 80% of the time (‘common’ transition) and displays 2 pink balls, giving a pink ball on 20% of trials (‘rare’ transition). C. The purple and pink balls have different values that dynamically change throughout the game. The value of the ball is defined as the probability of it being a ‘good ball’, i.e., one that remains intact after firing (rewarding trial), or a ‘dud ball’ (non-rewarding trial) that explodes shortly after being fired, and therefore cannot reach the diamond. D. We included 2 drifting reward probabilities (A, B) that quantitively differed on various metrics. Participants were randomly assigned a reward drift set at each block leading to four distinct drift set combinations (A-A, A-B, B-A, B-B).

Figure and legend reproduced with permission from Donegan, Brown (7) published in Communications Psychology under the CC-BY-4.0 license (https://creativecommons.org/licenses/by/4.0/).

## **Digital questionnaire response time – Cognitive processing speed**

Digital questionnaire response time is a passive measure of cognitive processing speed based on the time it takes a participant to respond to each individual item in a (set of) questionnaire(s). Item-level timestamps are extracted for each questionnaire or EMA item set to calculate the time difference between each consecutive timestamp and obtain item-level response times. As careless responding or interruptions can lead to response times that are artificially long or short, a two-tiered quality control approach is used as described in Teckentrup, Rosicka (8). First, single item response times that are longer than 900 seconds are removed before calculating the median response time across all items. Then all response times greater the median response time plus two standard deviations are removed. No lower threshold is applied.

This measure was validated in a prior paper in detail. In brief, across several samples DQRT was linked to task-based measures of processing speed (range *r*=0.29-0.39, all p<0.001) and working memory (range *r*=0.11-0.14, all p<0.001), supporting a cognitive characterization of DQRT as a measure of ‘cognitive processing speed’. Using an intraclass correlation (ICC; two-way mixed-effects model with absolute agreement, single rater) analysis, DQRT showed good reliability of *ICC* [*CI*]=0.85 [0.83 0.88] (*p*<0.001) for same-day assessments. For assessments with an interval ranging from one day to one week, reliability remained good, *ICC* [*CI*]=0.80 [0.75 0.84] (*p*<0.001), and this decreased to moderate to good *ICC* [*CI*]=0.76 [0.67 0.82] (*p*<0.001) for a lag of >1 week between assessments. and associations between Using longitudinal EMA assessments, DQRT showed a moderate reliability *ICC* [*CI*]=0.63 [0.55 0.71] (*p*<0.001). Intriguingly, associations between DQRT and task-based processing speed were higher within individuals (*r*=0.35) than between individuals (*r*=0.25).

# **REFERENCES**

1. Brooks ME, Kristensen K, Benthem KJv, Magnusson A, Berg CW, Nielsen A, et al. glmmTMB Balances Speed and Flexibility Among Packages for Zero-inflated Generalized Linear Mixed Modeling. The R Journal. 2017;9(2).

2. Benjamini Y, Hochberg Y. Controlling the False Discovery Rate: A Practical and Powerful Approach to Multiple Testing. Journal of the Royal Statistical Society Series B: Statistical Methodology. 1995;57(1):289-300.

3. Sheehan DV, Lecrubier Y, Sheehan KH, Amorim P, Janavs J, Weiller E, et al. The Mini-International Neuropsychiatric Interview (M.I.N.I.): the development and validation of a structured diagnostic psychiatric interview for DSM-IV and ICD-10. J Clin Psychiatry. 1998;59 Suppl 20:22-33;quiz 4-57.

4. Fleming SM, Ryu J, Golfinos JG, Blackmon KE. Domain-specific impairment in metacognitive accuracy following anterior prefrontal lesions. Brain. 2014;137(Pt 10):2811-22.

5. Fox CA, McDonogh A, Donegan KR, Teckentrup V, Crossen RJ, Hanlon AK, et al. Reliable, rapid, and remote measurement of metacognitive bias. Sci Rep. 2024;14(1):14941.

6. Daw ND, Gershman SJ, Seymour B, Dayan P, Dolan RJ. Model-based influences on humans' choices and striatal prediction errors. Neuron. 2011;69(6):1204-15.

7. Donegan KR, Brown VM, Price RB, Gallagher E, Pringle A, Hanlon AK, et al. Using smartphones to optimise and scale-up the assessment of model-based planning. Commun Psychol. 2023;1(1):31.

8. Teckentrup V, Rosicka AM, Donegan KR, Gallagher E, Hanlon AK, Gillan CM. Digital questionnaire response time (DQRT): A ubiquitous and low-cost digital assay of cognitive processing speed. Behav Res Methods. 2025;57(7):200.
